# Merohedral icosahedral M_48_ (M = Co^II^, Ni^II^) cage clusters supported by thiacalix[4]arene†
†Electronic supplementary information (ESI) available: Scheme, magnetic susceptibility measurements, MALDI-TOF mass spectra, PXRD, TGA, FT-IR spectra, UV-vis-NIR spectra, additional figures and tables. CCDC 1526121 and 1526122. For ESI and crystallographic data in CIF or other electronic format see DOI: 10.1039/c8sc03193b


**DOI:** 10.1039/c8sc03193b

**Published:** 2018-09-12

**Authors:** Dantong Geng, Xu Han, Yanfeng Bi, Yucai Qin, Qiang Li, Liangliang Huang, Kun Zhou, Lijuan Song, Zhiping Zheng

**Affiliations:** a College of Chemistry , Chemical Engineering and Environmental Engineering , Liaoning Shihua University , Fushun 113001 , P. R. China . Email: biyanfeng@lnpu.edu.cn ; Email: lsong56@263.net; b Shenzhen Grubbs Institute and Department of Chemistry , Southern University of Science and Technology , Shenzhen , Guangdong 518000 , China . Email: zhengzp@sustc.edu.cn

## Abstract

Cage clusters are a discrete chemically and topologically diverse family of molecule-based functional materials. Presented here are two isostructural M_48_ (M = Co^II^ for **LSHU01**, Ni^II^ for **LSHU02**) cage clusters with a merohedral icosahedral cage structure featuring 12 M_4_-TC4A (H_4_TC4A, *p-tert*-butylthiacalix[4]arene) second building units as vertices and 18 asymmetric 5-(1*H*-tetrazol-1-yl)isophthalate ligands as faces. They are the highest-nuclearity cage compounds of Co^II^ and Ni^II^. The activated Co_48_ cage exhibited high selectivity in the sorption of C_3_H_8_ over CH_4_ under ambient conditions. Frequency response experiments indicated that the extrinsic voids and matrix interface of the activated crystalline samples are primarily responsible for the observed gas adsorption performance.

## Abstract 2

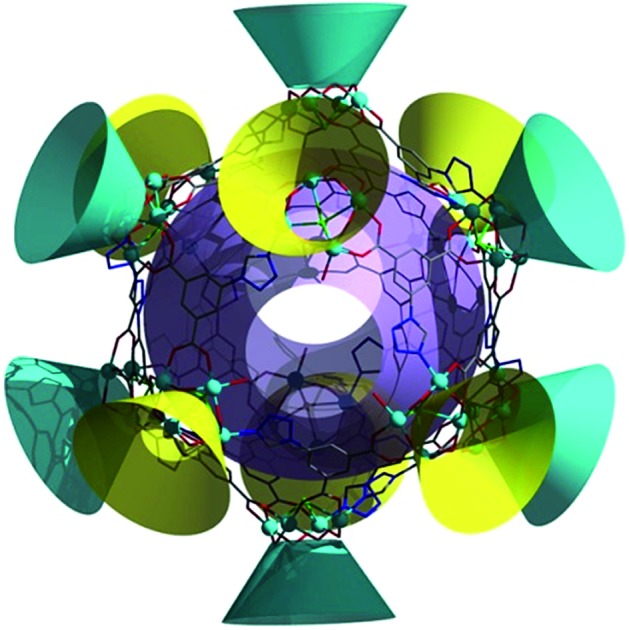
Highest nuclearity M_48_ (M = Co^II^ or Ni^II^) cage clusters have been constructed by [12+18] condensation of twelve M4-*p-tert*-butylthiacalix[4]arene second building units (as vertices) and eighteen asymmetric 5-(1*H*-tetrazol-1-yl)isophthalate ligands (as faces), showing a merohedral icosahedron-type framework.

## Introduction

Polyhedral coordination clusters (PCCs) are an intriguing class of compounds because of their structural aesthetics, interesting physical and chemical properties, and potential application as molecule-based functional materials.^1^–^4^ Numerous such species varying in both structure and composition have been reported. Although their nuclearities and overall structures generally cannot be predicted *a priori*, in many of such species smaller and structurally well-defined complex units are clearly recognizable.^5^–^10^ For example, the Mn_84_ torus can be formally built by alternating linear [Mn_3_O_4_] and cubic [Mn_4_O_2_(OMe)_2_] units;^11^ the record-setting Fe_64_ cage consists of Fe_8_ units linked by H_3_tea (triethanolamine) ligands,^12^ while the spheroidal Cu_36_ cluster can be formally constructed from metal–organic units.^13^,^14^ The structural modularity of such PCCs suggests the possibility of assembling even larger polynuclear species by using preformed cluster building units in combination with various bridging ligands, in a stepwise fashion or formally from a one-pot reaction.^15^–^17^ Efforts along this line have indeed produced much success. For example, the largest Co^II^ cluster known (Co_36_) can be viewed as an assembly of a cubane core of Co_12_ clamped by two identical Co_12_ wings *via* a 2,3-dicarboxypyrazine ligand.^18^

Typical calixarenes and their derivatives are used as multidentate ligands for assembly of cage clusters, *e.g.* Co_8_/Ni_8_ and Ni_24_/Co_24_ cage clusters, that have been reported by Atwood.^19^–^23^ In comparison, *p-tert*-butylthiacalix[4]arene (H_4_TC4A, Scheme S1a, ESI†) and its derivatives^24^–^26^ with four potentially bridging coordinating groups (S, SO, and SO_2_) and four OH groups form tetranuclear M^II^-thiacalixarene compounds that have been used as secondary building units (SBUs) with appropriate bridging ligands for the construction of more higher nuclearity PCCs including the highest nuclearity Co_32_ and Ni_40_ cages.^27^–^30^ Recently, these PCCs were also found to have new applications in molecule-based materials, and it was demonstrated that the obtained properties highly rely on the cluster shapes and components.^31^–^36^ One interesting observation is that when bridging units of an apparent structural symmetry were used, clusters of regular polyhedral structures including tetrahedral,^37^ cubic,^29^ and octahedral^27^,^28^,^32^–^34^,^38^–^45^ platonic solids were obtained. In comparison, the use of a less symmetric bridging ligand afforded an even larger cage cluster such as the largest known Ni_40_ cage containing 10 Ni_4_-TC4A units.^30^ Although the profound ligand effect is clear, how exactly the ligand dictates the assembly of the clusters eventually formed remains unclear. More such species with various compositions and shapes will help understand how these giant cage clusters may have been assembled. With such an understanding, we report here two isostructural M_48_ cage clusters of the general formula [M_48_(TC4A)_12_(L)_18_Cl_12_(H_2_O)_6_]·(+solvents) (**LSHU01**, M = Co; **LSHU02**, M = Ni) consisting of 12 M_4_-TC4A units and 18 deprotonated 5-(1*H*-tetrazol-1-yl)isophthalic acid (H_2_L, Scheme S1b, ESI†) ligands. They represent the largest known PCCs of Co^II^ and Ni^II^.

## Results and discussion

### Structure of M_48_ cage clusters

**Fig. 1:**
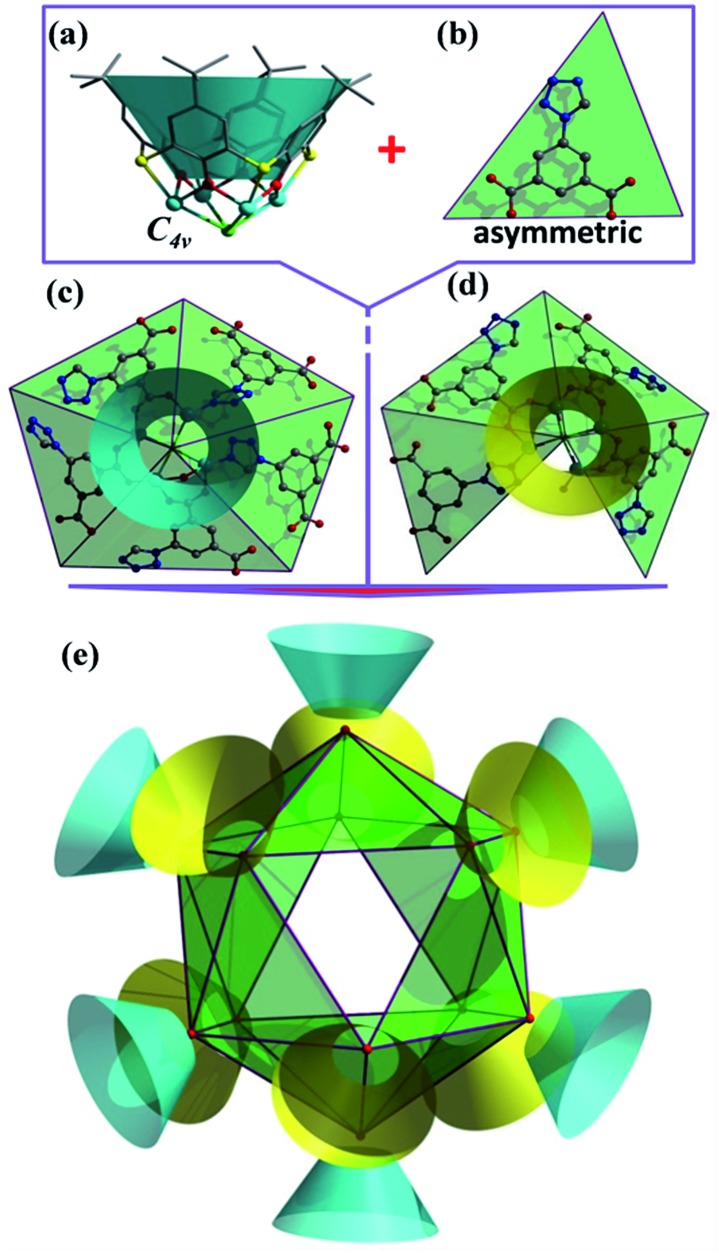
The structure of the M_48_ (M = Co, Ni) cage cluster determined by X-ray crystallography: (a) representations of the [(M_4_-TC4A)Cl] SBU with an approximate *C*_4v_ symmetry (all hydrogen atoms omitted for clarity), (b) the asymmetric 5-(1*H*-tetrazol-1-yl)isophthalate (L^2–^) ligand, (c) SBU-1 with five L^2–^ ligands, (d) SBU-2 with four L^2–^ ligands and (e) the M_48_ cage cluster showing the merohedral icosahedral arrangement of 12[(M_4_-TC4A)Cl] units. Color code: M turquoise, S yellow, Cl green, O red, N blue, C gray, TC4A in SBU-1 turquoise and in SBU-2 yellow, and merohedral icosahedron green.

Crystallographic studies (Table S1, ESI†) revealed that **LSHU01** and **LSHU01** are isomers and there are 2 M_4_-TC4A units, 3 L^2–^ ligands, 2Cl^–^ ions, and an aqua ligand in the asymmetric unit. The tetranuclear unit of M_4_-TC4A, with its TC4A ligand adopting a cone conformation, is capped at the bottom by μ_4_-Cl^–^ showing a *C*_4v_ symmetry (Fig. 1a). This unit structure is the same as previously reported.^24^ The expected metric values of the M–O, M–N, and M–S bond lengths (Table S2, ESI†), the charge-balanced composition as determined crystallographically, and the bond valence sum calculations (BVS) indicate that the cobalt and nickel ions are divalent. Both magnetic susceptibility measurements and XPS investigations are consistent with this conclusion (Fig. S1, ESI†).

Further structural analysis revealed subtle differences between the two M_4_-TC4A units in the asymmetric unit with the one (SBU-1, Fig. 1c) containing five L^2–^ ligands while the other (SBU-2, Fig. 1d) has four L^2–^ ligands. The L^2–^ ligands in SBU-1 exhibit two different coordination modes: three adjacent L^2–^ ligands each use one of its two carboxylate groups to bridge the four metal ions, while the remaining two L^2–^ ligands each use one of the N atoms in its tetrazolyl group to coordinate in a monodentate fashion as reported in the literature (Fig. S2, ESI†).^46^ In comparison, SBU-2 can be viewed as being derived from SBU-1 by replacing one of the two L^2–^ ligands that use the tetrazolyl N atom for coordination with an aqua ligand. SBU-1 and SBU-2 units, 6 of each kind, are organized into an icosahedron with the tetranuclear units occupying its 12 vertices (Fig. 1e). A more careful analysis of the structure revealed that the SBU-2 units are in an octahedral arrangement, while the SBU-1 units are in a chair-like arrangement, much like the more stable conformation of cyclohexane. Unlike in the arrangement of the SBU-1 units, the three adjacent SBU-2 units are not directly linked by an L^2–^ ligand. Rather, they are hinged by three L^2–^ ligands to form a metallamacrocycle with three aqua ligands, one on each SBU-2 unit, disposed within the ring structure (Fig. S3, ESI†). As such, the overall structure of the cage cluster can be best described as a merohedral convex icosahedron with 18 triangular faces rather than a regular icosahedron with 20 triangular faces (Fig. 1e). We note that this structure type is rarely observed in metal–organic systems despite its common existence in pure inorganic compounds including B_12_, Keggin-type polyoxometalates, and metal-centered endohedral clusters.^47^–^50^ With the assembly of UO_2_^2+^ and calix[5]arene-carboxylate being viewed as a dodecahedron,^51^ the present M_48_ cage clusters complete the five structural types of platonic solids in metal-calixarene chemistry (Fig. S4, ESI†).^19^,^24^–^26^ MALDI-TOF mass spectra confirmed the presence of the Co_48_ cluster (Fig. S5, ESI†).

**Fig. 2:**
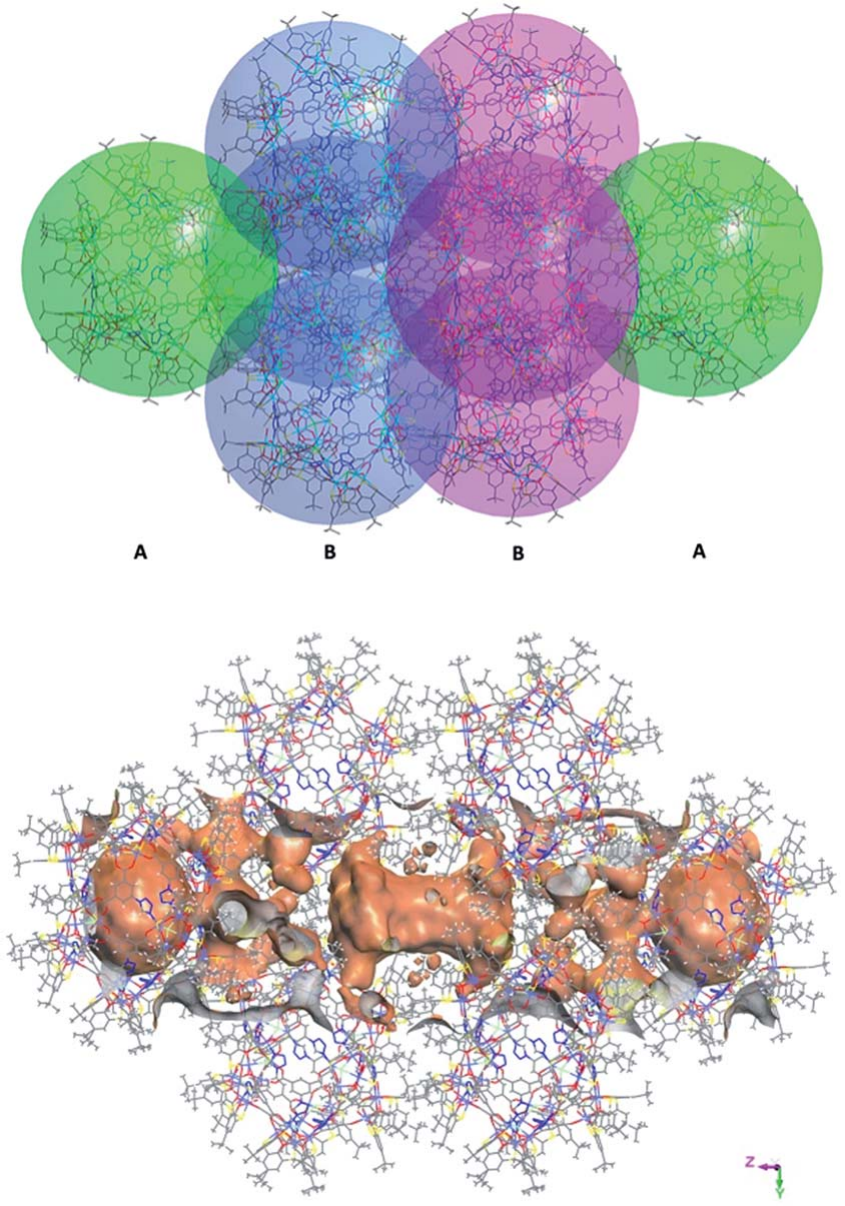
View of packing mode of the cages (up) and surface plot of the pores formed by arrangement in/between the cages (bottom).

The cage cluster has an outside dimension of 37.3 × 35.8 × 33.8 Å^3^ (
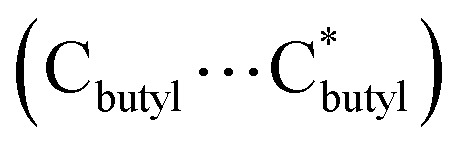
) with an inner cavity (Cl···Cl*) of 22.3 × 20.8 × 20.8 Å^3^; the latter is *ca.* 1.5 times larger than that of the Ni_40_ cage,^30^ although the former is similar. The solvent-accessible voids of **LSHU01** and **LSHU02** were estimated to be 54 653.5 Å^3^/64 029.6 Å^3^ per cell 96 152.0 Å^3^ and 106649.0 Å^3^, respectively, corresponding to 56.8%/60.0% of the total crystal volume. It is plausible that the different estimated solvent-accessible voids between the two isostructural cage clusters are due to the presence of different solvent molecules as well as the different collecting temperatures of the crystallographic data. That the hollow cages have two opposite triangle windows between two metallomacrocyclic rings partly occupied by aqua ligands is interesting. The edge of the triangle is *ca.* 7.6 Å with an in-circle diameter of *ca.* 5.4 Å (
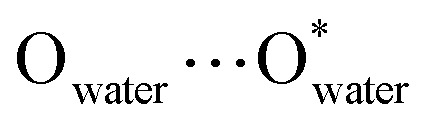
, Fig. S3, ESI†). Furthermore, irregular pockets between three cages are also observed with dimensions ranging from 4.0 to 11.7 Å (
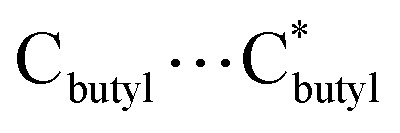
, Fig. S6, ESI†). By packing the cages along the *c* axis, one can find irregular channels with permanent pores (pore A, within cages) and packing pores (pore B, between adjacent cages) in ABBA mode (Fig. 2, S7 and S8, ESI†). The porous structure is expected to be useful for the uptake of gas molecules upon activation by desolvation.

### Stability and porosity

**Fig. 3:**
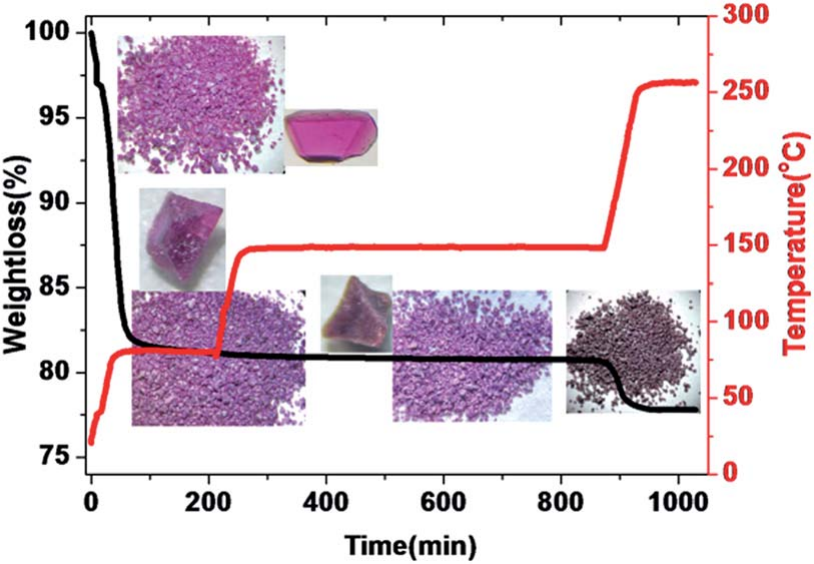
Temperature programmed desorption (TPD) of **LSHU01** (for example, heating rate 3 °C min^–1^ in a vacuum and maintained at 80 °C for 3 hours, 150 °C for 10 hours and 250 °C for 1 hour). Inset: crystal pictures and morphology corresponding to different states. The samples were placed at ambient temperature for several hours before the TPD experiment.

Both PXRD and thermogravimetric analysis indicated that **LSHU01** and **LSHU02** lost lattice solvent molecules between room temperature and 80 °C under vacuum (Fig. S9–S11, ESI†). Negligible weight loss in the temperature range of 80–150 °C for **LSHU01** suggests complete removal of organic solvents and maintenance of structural integrity (Fig. 3 and S11–S13, ESI†). Weight loss in the range of 150–250 °C can be attributed to loss of the coordinated water molecules and Cl atoms (calc: 3.3%, found: 3.1%).

In order to assess the porosity of the M_48_ cages, argon adsorption measurements were performed at 87 K using **LSHU01′** and **LSHU01′′** (**LSHU01** activated at 80 °C and 150 °C, respectively), for example **LSHU01** and **LSHU02** are isostructural and the cage clusters showed similar outside dimensions and inner cavities (Fig. S14, ESI†). The gas sorption showed a non-reversible type-I isotherm with large hysteresis upon desorption of **LSHU01′**. Such a behavior is probably due to the presence of the voids of the cage cluster and the extrinsic voids between cages as evidenced by the broad pore size distributions (PSDs) (0.50–1.10 nm with two peaks at *ca.* 0.51 nm and 0.65 nm, respectively, Fig. S14, inset†). A typical reversible type-I isotherm was obtained using **LSHU01′′**; its PSD is similar to that of **LSHU01′** but the decrease of surface areas at 0.65 nm suggested different microstructures of these two activated samples. The observation of the PSD at *ca.* 0.51 nm that can be ascribed to the cage windows for both **LSHU01′** and **LSHU01′′** suggested the undamaged framework of cage clusters after higher temperature evacuation. For **LSHU01′**, the Langmuir and Brunauer–Emmett–Teller (BET) surface areas are estimated to be 803.09 m^2^ g^–1^ and 582.02 m^2^ g^–1^, while for **LSHU01′′** the corresponding values are 607.95 m^2^ g^–1^ and 438.55 m^2^ g^–1^, respectively. The decreases in micropore areas (from 411.94 to 360.88 m^2^ g^–1^, 12.39%), external surface areas (from 170.08 to 77.67 m^2^ g^–1^, 54.33%), and micropore volume (from 0.1776 to 0.1600 cm^3^ g^–1^, 9.91%) for **LSHU01′′** with respect to **LSHU01′** are attributed to the decrease of free space between cages caused by the more compact packing of cages evacuated at higher temperature, consistent with Horvath–Kawazoe (HK) and Barrett–Joyner–Halenda (BJH) absorption PSDs (Fig. S15 and S16, ESI†).

### Gas absorption and separation

**Fig. 4:**
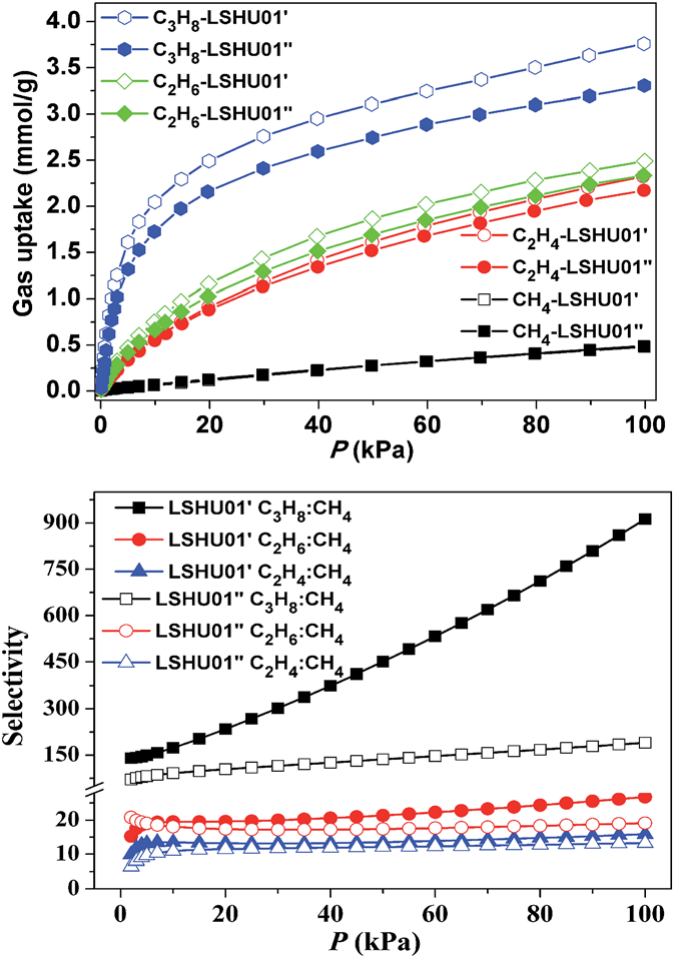
CH_4_, C_2_H_4_, C_2_H_6_ and C_3_H_8_ adsorption isotherms at 295 K (up) and gas mixture adsorption selectivity predicted by IAST at 295 K for **LSHU01′** and **LSHU01′′** (*P*: 1–100 kPa, gas ratios: 50 : 50, bottom).

The CH_4_, C_2_H_4_, C_2_H_6_, C_3_H_8_, and CO_2_ uptake experiments were conducted at 295 K in the pressure range of 0–100 kPa using **LSHU01′** and **LSHU01′′** (Fig. 4 right and Fig. S17–S23, ESI†). For CH_4_, the absorption isotherms are almost identical at about 0.49 mmol g^–1^ for **LSHU01′** and **LSHU01′′** at 1 bar. The adsorption capacity of **LSHU01′** at 1 bar for C_2_H_4_, C_2_H_6_, C_3_H_8_, and CO_2_ is 2.32, 2.49, 3.75, and 1.77 mmol g^–1^, respectively, all being higher than the corresponding values (2.17, 2.33, 3.31, and 1.67 mmol g^–1^) obtained with the use of **LSHU01′′** (Fig. S23, ESI†). The calculated isosteric heat (*Q*_st_) of adsorption at zero loading is 26.27 and 39.50 kJ mol^–1^ for CH_4_, 20.53 and 25.79 kJ mol^–1^ for C_2_H_4_, 23.51 and 32.15 kJ mol^–1^ for C_2_H_6_, and 38.16 and 47.76 kJ mol^–1^ for C_3_H_6_ by virial equation and dual-site Langmuir Freundlich (DSLF) equation fitting, respectively.^52^–^55^ The values remained essentially unchanged for the loading of CH_4_, C_2_H_4_, C_2_H_6_, and CO_2_ using the virial method. The values by the DSLF method are slightly higher in the lower gas uptake region but approached the values obtained using the virial method at 1 bar. The obtained *Q*_st_ plots for C_3_H_8_ using virial and DSLF equations are almost identical and can be used to describe the whole absorption isotherms (Fig. S20, ESI†). The difference in the calculated *Q*_st_ values using the virial equation and the DSLF method indicated the dynamic absorption behavior of the discrete Co_48_ cages.^55^

For binary mixtures composed of equimolar C_2_-hydrocarbons or CO_2_ and CH_4_, the selectivity is below 30 for both **LSHU01′** and **LSHU01′′** (Fig. 4 right), which is comparable to many systems previously reported.^56^–^60^ However, the initial selectivity for the C_3_H_8_/CH_4_ mixture was 140.1 and 70.5 and reached 912.6 and 189.8 at 295 K and 1 bar for **LSHU01′** and **LSHU01′′**, respectively (Fig. S22 and S23, ESI†). It is highlighted that the selectivity for C_3_H_8_/CH_4_ under ambient conditions of **LSHU01′** is about 30 fold that of the closed Ni_40_ cage^30^ and the best performance among metal–organic polymers (Table S3, ESI†). The extremely high selectivity values for C_3_H_8_/CH_4_ strongly suggest that it is feasible to separate the pair in a vacuum swing adsorption process using the activated Co_48_ sample as an adsorbent.

### Frequency response

**Fig. 5:**
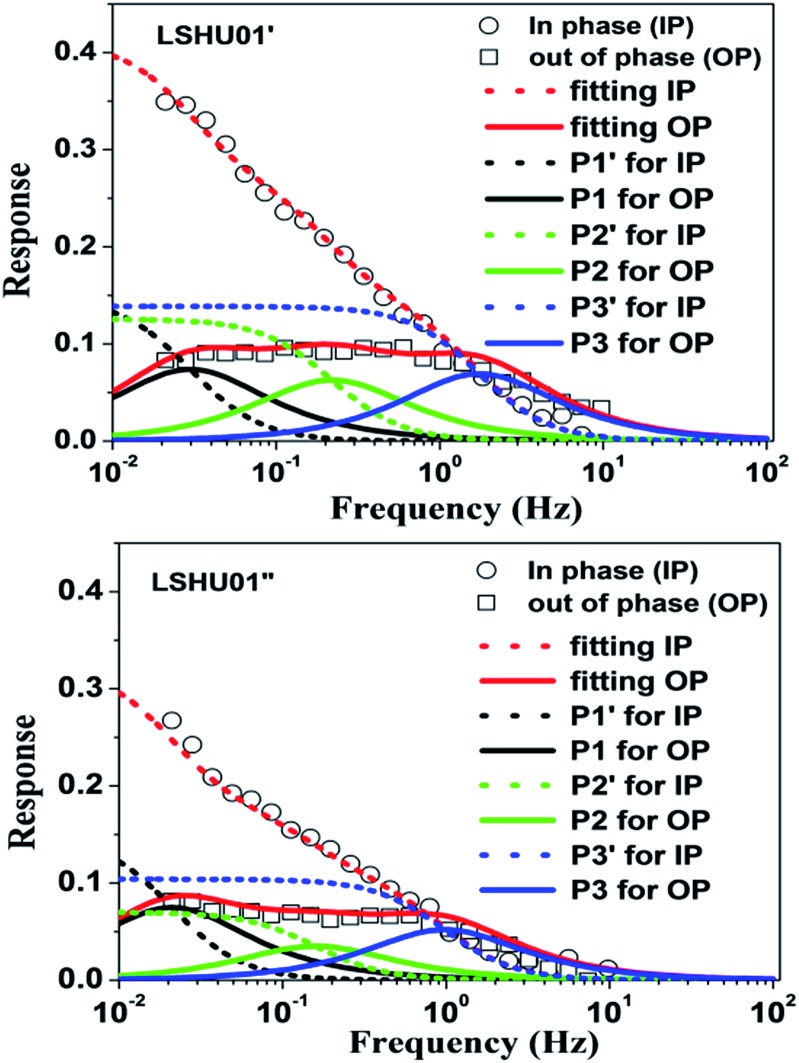
Frequency response (FR) spectra of C_3_H_8_ for **LSHU01′** (up) and **LSHU01′′** (bottom) at 0.133 kPa, 0–10 Hz.

To understand why **LSHU01′** showed higher selectivity than **LSHU01′′** in the adsorption of C_3_H_8_ over CH_4_ at 295 K, frequency response (FR) spectra of C_3_H_8_ and CH_4_ were obtained. Both in-phase (IP) and out-of-phase (OP) FR signals of C_3_H_8_ can be detected using both **LSHU01′** and **LSHU01′′** (Fig. 5), but not for CH_4_ (Fig. S24, ESI†). The OP curves of C_3_H_8_ spectra, well fitted by the Yasuda sorption theoretical model,^61^,^62^ correspond to three adsorption processes (P1–P3) for **LSHU01′** and **LSHU01′′**, which can be ascribed to the absorption in the molecular cages, between cages, and on the interface of the matrix from low frequency to high frequency, respectively. The reduction of the response intensity of IP and OP for **LSHU01′′***versus***LSHU01′** indicates the lower absorption of C_3_H_8_ molecules, which is consistent with the decrease in BET surface areas and the C_3_H_8_-absorption experiments. Furthermore, the fitted OP response peaks P1–P3 are shifted to lower frequency (from 0.03 to 0.02 Hz for P1, from 0.22 to 0.16 Hz for P2, and from 1.74 to 0.97 Hz for P3) for **LSHU01′′** with respect to **LSHU01′**, pointing to the lower absorption rate of C_3_H_8_ molecules.^55^,^63^ The observations detailed above together indicate that the free space between the cages and matrix interface plays an important role in the uptake and separation of C_3_H_8_ over CH_4_ under ambient conditions. It is also concluded that the absorption properties are sensitively dependent on the pore size, the moderate surface areas in accordance with gas kinetic diameters, and/or the polarizability of the gas molecules.^64^–^66^

## Conclusions

In summary, we have obtained and structurally characterized two record-setting high-nuclearity M_48_ (M = Co, Ni) cage clusters. It has been shown that higher-nuclearity Co and Ni cages can be built by using M_4_-TC4A SBUs and asymmetric ligands. The merohedral icosahedron-type structures with large pores and windows allow us to investigate gas sorption properties of the crystalline materials. The activated Co_48_ crystals were proved to be a competitive candidate for separating C_3_H_8_ from CH_4_. The activation temperature for Co_48_ crystals has a profound effect on the extrinsic voids between the cage clusters which are believed to be primarily responsible for the observed absorption properties and selectivity. Efforts aimed at the synthesis of even higher-nuclearity cage clusters are ongoing.

## Experimental section

### Materials and measurements

*p-tert*-Butylthiacalix[4]arene (H_4_TC4A)^67^ and 5-(1*H*-tetrazol-1-yl)isophthalic acid (H_2_L)^68^ were synthesized according to a literature method, respectively. Other reagents were purchased from commercial sources and used as received. TGA was performed on a Perkin Elmer Pyris1 TGA thermo-gravimetric analyzer. FT-IR spectra using KBr pellets were taken on a Perkin Elmer Spectrum GX spectrometer. UV-vis spectra were recorded on an Agilent Cary 5000 spectrometer. MALDI-TOF mass data were collected on a Bruker Autoflex III Smartbeam MALDI-TOF mass spectrometer. CHNS elemental analysis was performed on an EAI CE-440 instrument. Magnetic susceptibility measurements were performed on a Quantum Design MPMS XL-5 SQUID system in the temperature range of 2–300 K, and diamagnetic corrections for the sample and sample holder were applied. X-ray photoelectron spectroscopic (XPS) measurements were carried out with an ESCALAB 250Xi using a monochromatic Al Kα X-ray source (1486.6 eV).

### Synthesis of **LSHU01** and **LSHU02**

H_2_L was added to a suspension of MCl_2_·6H_2_O (M = Co, Ni) (0.95 g, 0.4 mmol) and H_4_TC4A (0.09 g, 0.125 mmol) in a *m* : *n* (v/v) CHCl_3_–CH_3_OH mixed solvent (total 10 mL, *m* = 8, *n* = 2 for Co; *m* = 5, *n* = 5 for Ni) with stirring for 10 min and then transferred into a 20 mL Teflon-lined autoclave which was kept at 130 °C for 3 days and then slowly cooled to room temperature at about 4 °C h^–1^. Red/green crystals were isolated by filtration and then washed with *m* : *n* CHCl_3_–CH_3_OH and dried in a vacuum at room temperature. Yield (0.094 g and 0.043 g): *ca.* 55% and 26% with respect to H_4_TC4A for **LSHU01** and **LSHU02**, respectively. Elemental analysis: calculated (%) for [M_48_(C_40_H_44_S_4_O_4_)_12_(C_9_H_4_N_4_O_4_)_18_Cl_12_(H_2_O)_6_]: M = Co, C 44.76, H 3.82, N 6.25, S 9.53; M = Ni, C 44.79, H 3.82, N 6.25, S 9.54; found: M = Co, C 44.60, H 3.96, N 6.15, S 9.38, found: M = Ni, C 44.60, H 3.96, N 6.15, S 9.38 (after being dried in a vacuum at 80 °C).

### Single crystal X-ray diffraction

The intensity data were recorded on a Bruker D8 QUEST system with Mo-K*α* radiation (*λ* = 0.71073 Å). The crystal structures were solved by means of direct methods and refined by employing full-matrix least squares on *F*2 (SHELXTL-2014).^69^ Even the low temperature data set obtained at about 100 K for the compound **LSHU01** reveals severely disordered solvents within the lattice interstices that are difficult to interpret thus complicating efforts to give precise estimates of the molecular formula. The diffraction data were treated by the “SQUEEZE” method as implemented in PLATON (see ESI† for details).^70^ All non-hydrogen atoms were refined anisotropically, and hydrogen atoms of the organic ligands were generated theoretically onto the specific atoms and refined isotropically with fixed thermal factors. Since the crystals do not diffract very well at high angles due to the structural disorder, the determined 2*θ* is 22.0141° and 20.1027° for **LSHU01** and **LSHU02**, respectively. The *R* factors in the final structural refinement are also relatively large but typical of such systems.^27^,^29^,^45^ Refinement parameters and crystallographic data, selected bond distances and BVS calculations for **LSHU01** and **LSHU02** are shown in Tables S1 and S2 in the ESI,† respectively. CCDC ; 1526121 and ; 1526122 contain the supplementary crystallographic data of **LSHU01** and **LSHU02** for this paper, respectively.

### Gas adsorption experiments

Ultra-high-purity grade gas (>99.99) was used throughout the adsorption experiments. The measured sorption isotherms have been recorded at least two times to confirm the reproducibility within experimental errors. About 150 mg of methanol solvent-exchanged Co_48_ samples were activated at 80 °C and 150 °C for 10 hours according to the TPD experiment, respectively. Low-pressure gas sorption experiments are carried out on Micromeritics ASAP 2020M automatic volumetric instruments for Ar and on an Intelligent Gravimetric Analyser (IGA-003 Hiden Analytical Ltd., Warrington, UK.) for CO_2_, CH_4_, C_2_H_4_, C_2_H_6_ and C_3_H_8_, respectively. Buoyancy corrections for the samples were applied for gravimetric measurements. Desorption was achieved by placing the samples under a dynamic vacuum at 80 °C or 150 °C for three hours, respectively. Ar isotherms were measured using a liquid argon bath (87 K). Other gas isotherms, *e.g*. for CO_2_, CH_4_, C_2_H_4_, C_2_H_6_ and C_3_H_8_, were measured at 273 K and 295 K, respectively. The specific surface areas are determined using the Brunauer–Emmett–Teller and Langmuir equations from the Ar sorption data. The pore size distribution was obtained from the DFT, HK, and BJH models in the Micromeritics ASAP 2020 software package based on the Ar sorption at 87 K.

### Frequency response measurements

Frequency response measurements were carried out on a high-accuracy differential Baratron pressure transducer (MKS 698A11TRC). The frequencies were controlled using an on-line computer, which was also used for the acquisition of the pressure data from the Baratron. An accurate amount of sample (*ca.* 30 mg) was scattered in a plug of glass wool and degassed under a high vacuum (<10^–3^ Pa) at 80 °C or 150 °C for 6 h. Prior to the measurement, the sorbate vapor from the supply side of the vapor reservoir was admitted to the sorption chamber and equilibrated over the sample at a certain pressure.^63^

## Conflicts of interest

There are no conflicts to declare.

## Supplementary Material

Supplementary information

Crystal structure data
