# Supplementary material for: Merohedral icosahedral M48 (M = CoII, NiII) cage clusters supported by thiacalix[4]arene
Source: Chem Sci. 2018 Sep 12;9(45):8535–41. doi: 10.1039/c8sc03193b (PMC6251337; doi:10.1039/c8sc03193b)
Supplement: Supplementary file 1 [file SC-009-C8SC03193B-s001.pdf]

Electronic Supplementary Material (ESI) for Chemical Science.
This journal is © The Royal Society of Chemistry 2018

Supporting Information

Merohedral Icosahedral M_{48} (M = Co^{II}, Ni^{II}) Cage Clusters Supported by Thiacalix[4]arene

Dantong Geng,[a‡] Xu Han[a‡] Yanfeng Bi,[*a] Yucai Qin,[a] Qiang Li,[a] Liangliang Huang,[a] Kun Zhou,[a] Lijuan Song,[*a] and Zhiping Zheng*[ab]

[a]College of Chemistry, Chemical Engineering and Environmental Engineering, Liaoning Shihua University, Fushun 113001, China
[b]Shenzhen Grubbs Institute and Department of Chemistry, Southern University of Science and Technology, Shenzhen, Guangdong 518000, China
*E-Mail: biyanfeng@lnpu.edu.cn; lsong56@263.net; zhengzp@sustc.edu.cn

Note added after first publication
25th September 2018: This electronic supplementary information file replaces that uploaded on 12th September 2018 in which affiliation *b* and the email address for Zhiping Zheng hadn't been updated.

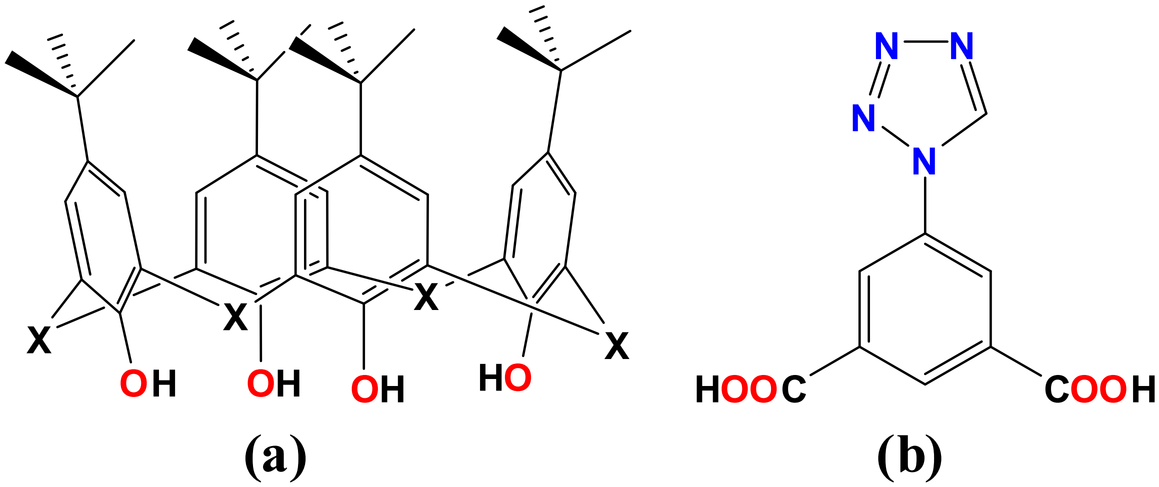

Scheme S1. (a) X = S for *p-tert*-Butylthiacalix[4]arene (H_4TC4A); X = SO, *p-tert*-Butylsulfinylcalix[4]arene; X = SO_2 for *p-tert*-Butylsulfonylcalix[4]arene; (b) 5-(1H-tetrazol-1-yl)isophthalic acid (H_2L).

Determine divalent nature of metal ions in LSHU01 and LSHU02

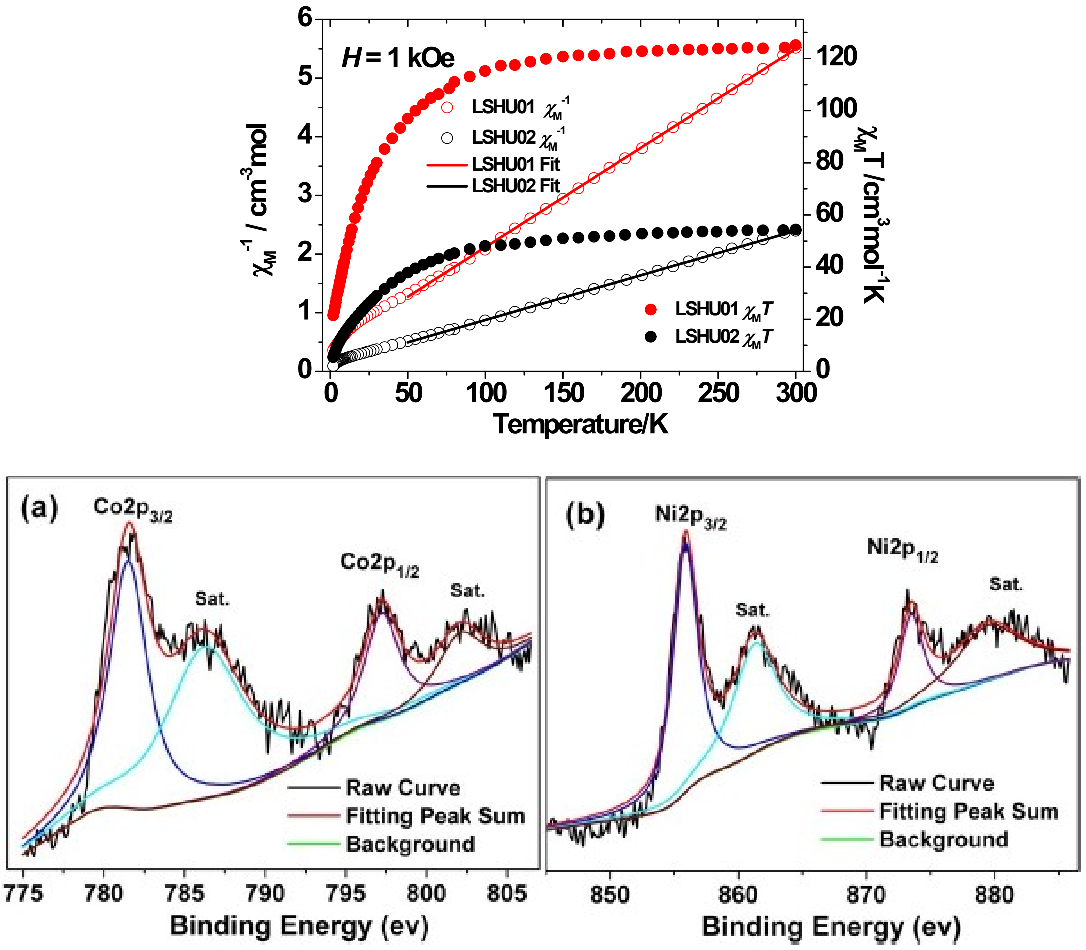

Fig. S1 Magnetic susceptibility measurements for **LSHU01** and **LSHU02** were performed on the fresh samples (the samples were isolated from mother liquids, wiped off the surface solvents, and encapsulated in sample holders quickly) with an applied magnetic field of 1KOe (up). The $\chi_{M}T$ value decreases gradually from 125.12 cm^3 mol^{-1} K/54.39 cm^3 mol^{-1} K at room temperature to 21.44 cm^3 mol^{-1} K/5.47 cm^3 mol^{-1} K at 2 K for **LSHU01** and **LSHU02**, respectively, showing the M^{II} nature of metal ions. Fitting the experimental data ranging from 50~300 K to Curie-Weiss law give Curie constant (C) being of 131.58 cm^3 mol^{-1} K/59.17 cm^3 mol^{-1} K and Weiss constant (θ) being of -15.26 K/-25.34 K, which indicate of antiferromagnetic interaction between the metal centers and/or the spin–orbit coupling effect of M^{II} ions [S1];

The high resolution of Co 2p XPS peak (bottom a) and Ni 2p XPS peak (bottom b). Both magnetic susceptibility measurements and XPS investigations indicated the divalent of metal ions in **LSHU01** and **LSHU02.**

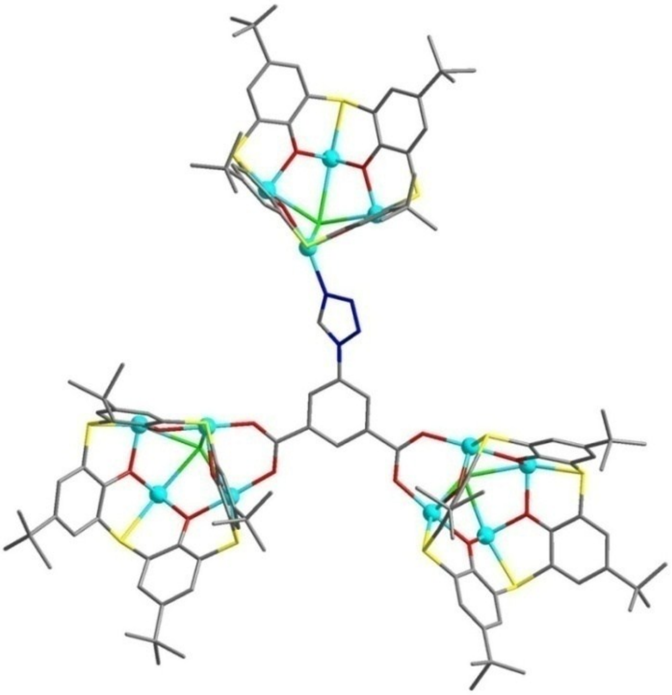

Fig. S2 The coordination mode of the L ligand

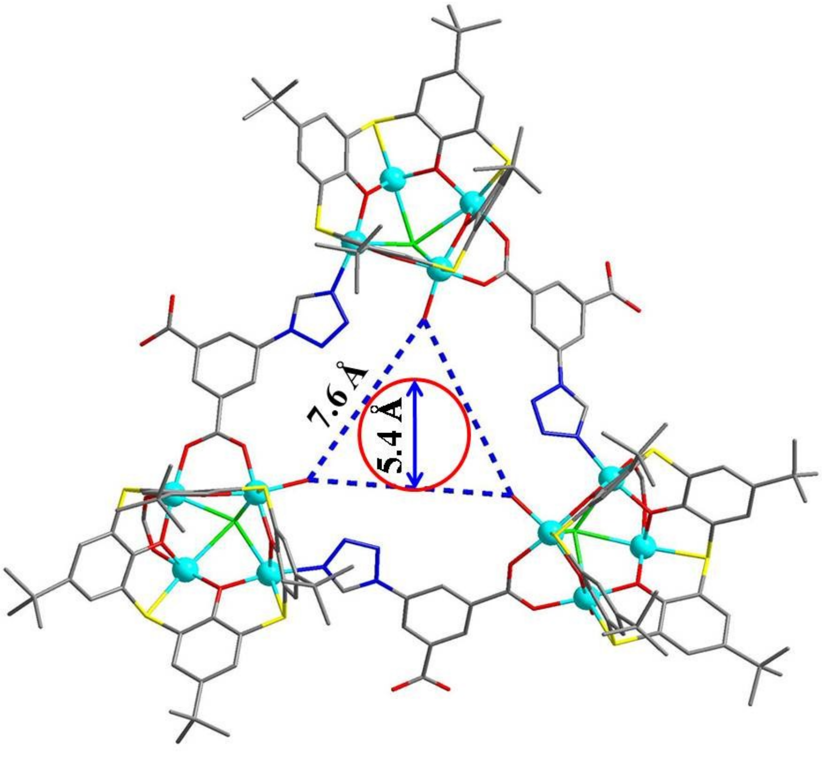

Fig. S3 Representation of the metallamacrocycle ring (window of the cage) partly occupied by coordinated water molecules. The triangle edge length is ca. 7.6 Å with incircle diameter ca. 5.4 Å based on $O_{water}\cdots O_{water}{}^{*}$

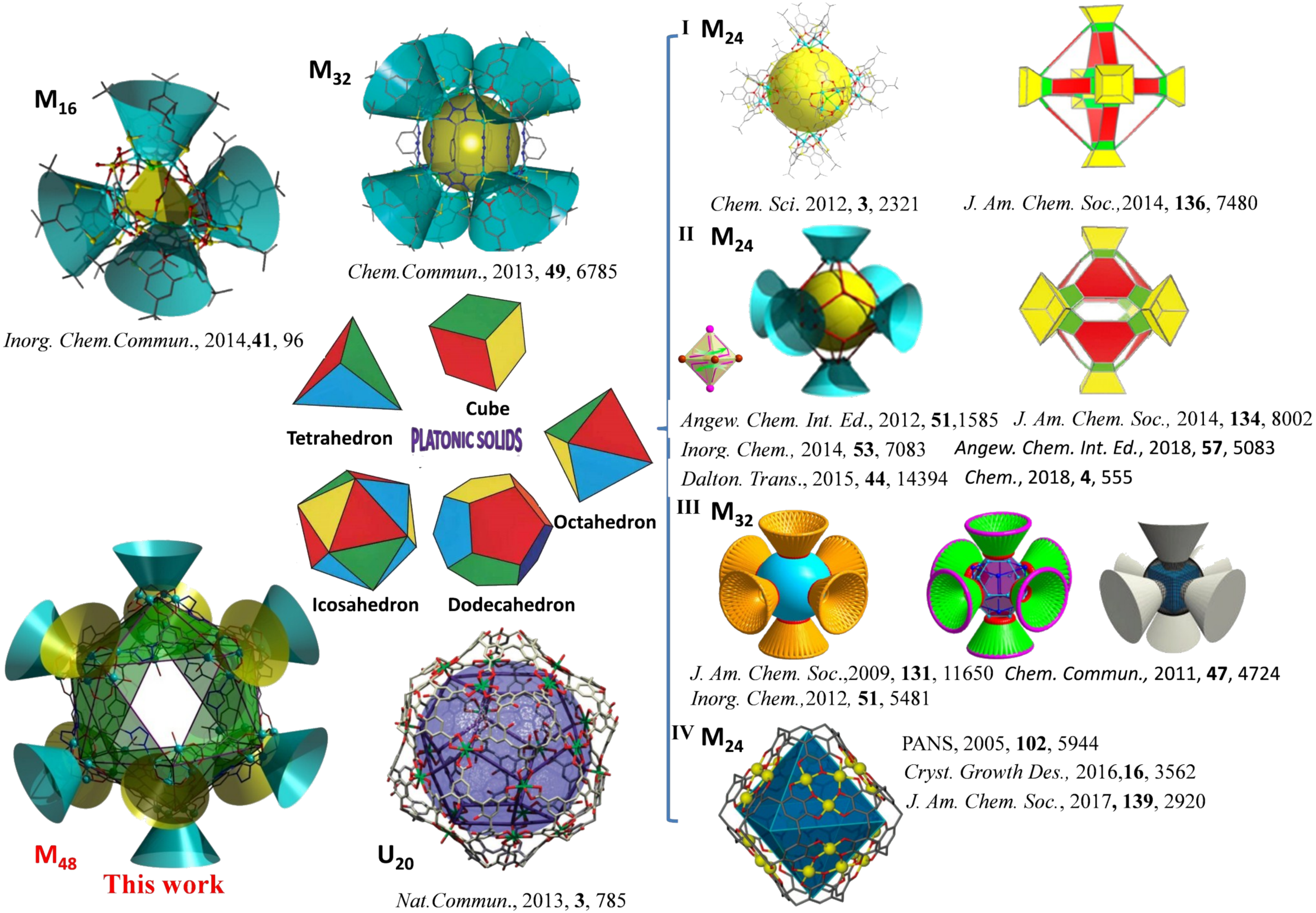
M16
Inorg. Chem.Commun., 2014,41, 96
M32
Chem.Commun., 2013, 49, 6785
Tetrahedron
Cube
PLATONIC SOLIDS
Octahedron
Icosahedron
Dodecahedron
M48
This work
U20
Nat.Commun., 2013, 3, 785
I M24
Chem. Sci. 2012, 3, 2321
J. Am. Chem. Soc.,2014, 136, 7480
II M24
Angew. Chem. Int. Ed., 2012, 51,1585
J. Am. Chem. Soc., 2014, 134, 8002
Inorg. Chem., 2014, 53, 7083
Angew. Chem. Int. Ed., 2018, 57, 5083
Dalton. Trans., 2015, 44, 14394
Chem., 2018, 4, 555
III M32
J. Am. Chem. Soc.,2009, 131, 11650
Chem. Commun., 2011, 47, 4724
Inorg. Chem.,2012, 51, 5481
IV M24
PANS, 2005, 102, 5944
Cryst. Growth Des., 2016,16, 3562
J. Am. Chem. Soc., 2017, 139, 2920

Fig. S4 Calixareme supported cage clusters showing platonic solids arrangements of metal ions (M= Co or Ni).

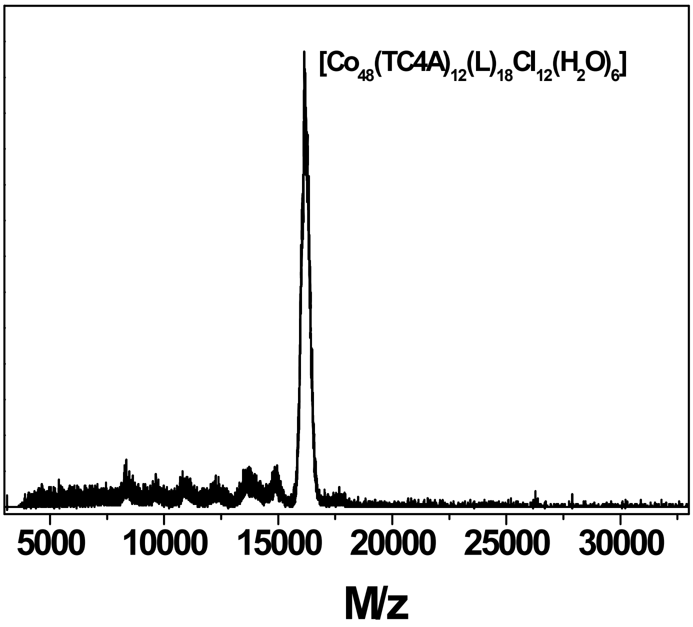

Fig. S5 MALDI-TOF mass spectra of **LSHU01'** showing the mass corresponding to [$Co_{48}(TC4A)_{12}(L)_{18}Cl_{12}(H_2O)_6$](m/Z=16144.92). The m/Z peaks below16000 may attributed to the deposition of Co_{48} cages by hyperacoustic in process of measurement that cannot be properly assigned. The samples were activated at 80°C in vacuum for six hours and dissolved in $CHCl_3$-THF(1:1) mixture.

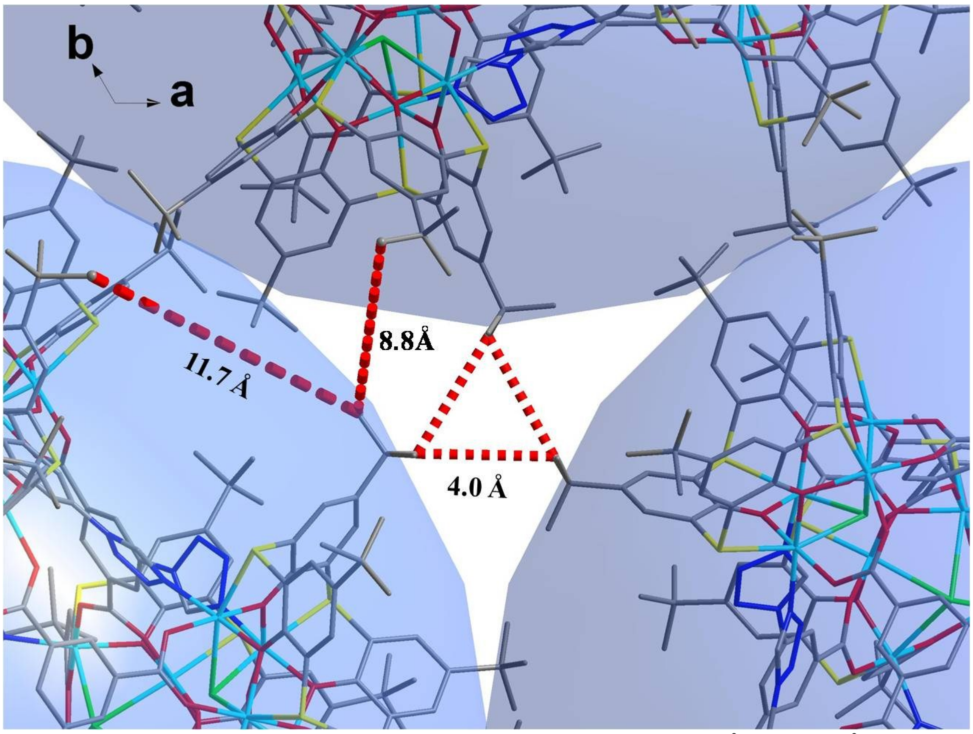

Fig. S6 Pockets among three cages with dimensions from 4.0 Å to 11.7Å showing irregular size pore distributions

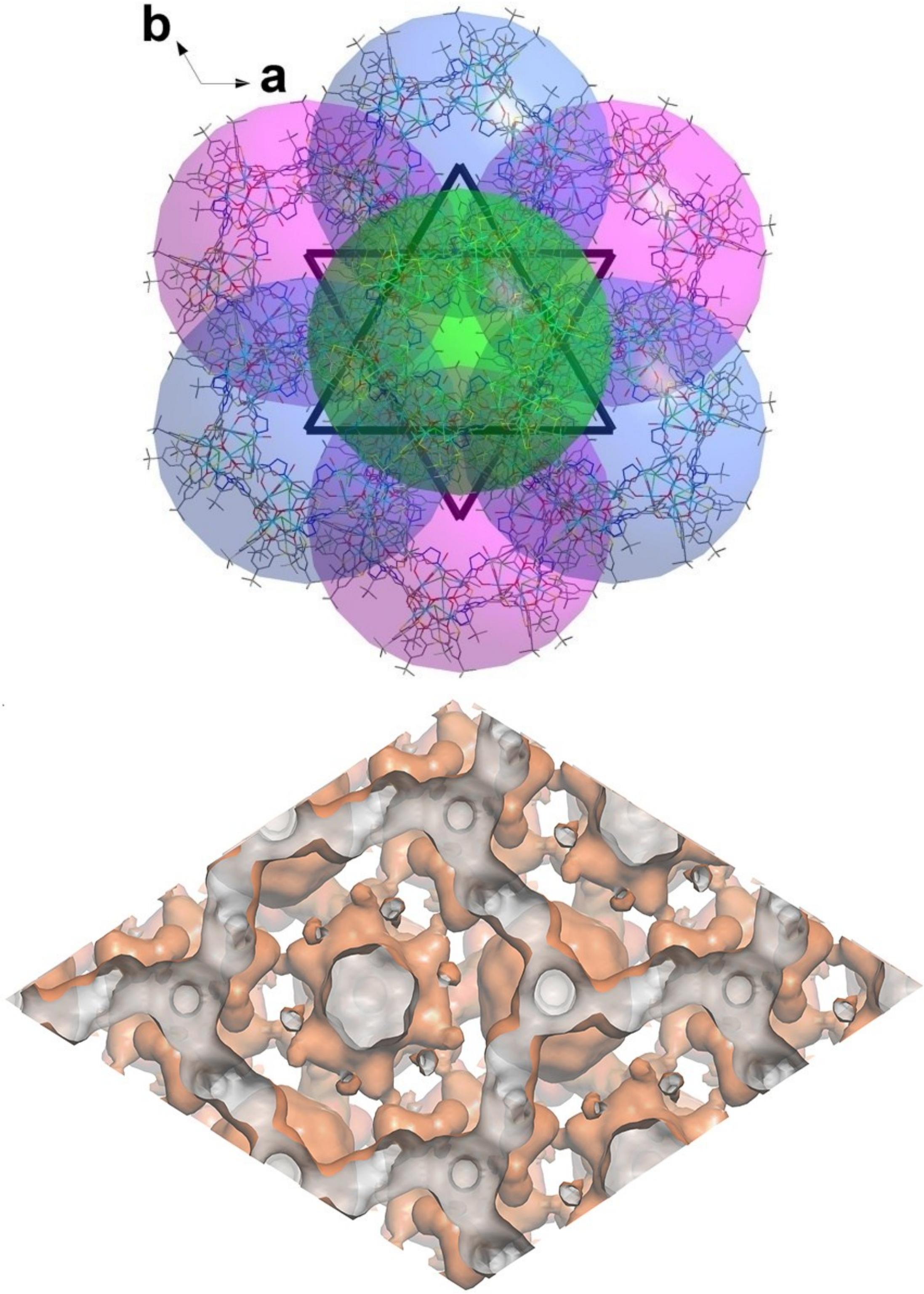

Fig. S7 Top view of the packing mode of the cages (up) and surface plot of LSHU01 showing the multiporous structures(bottom).

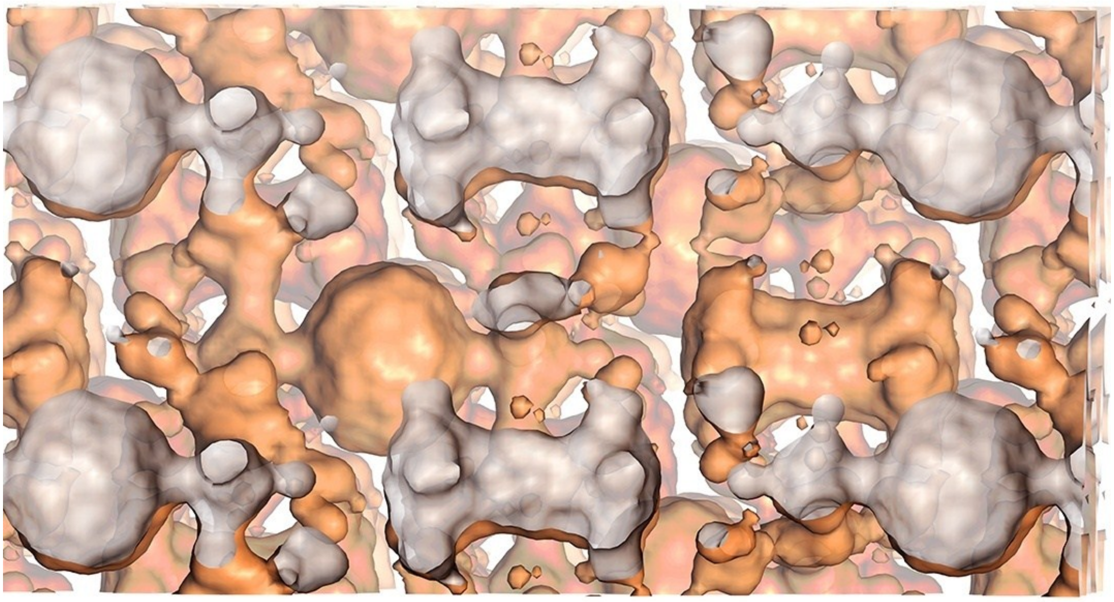

Fig. S8 The surface view of LSHU01 of packing mode showing the connected irregular channels

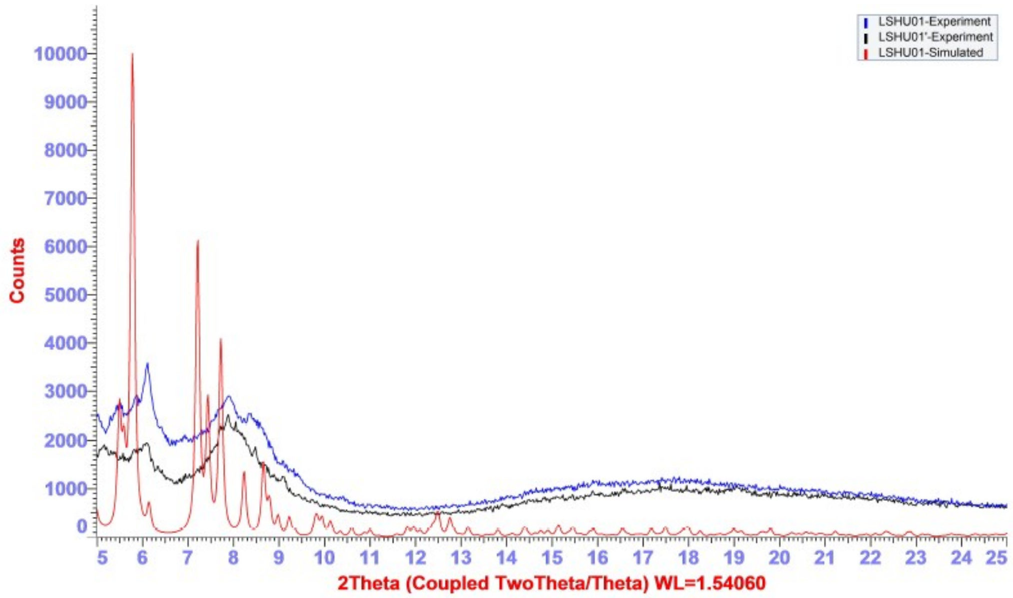

Fig. S9 Powder XRD patterns of **LSHU01** and **LSHU01'** shows that the crystals lose crystallinity at ambient conditions and the cage structures may maintained after activated at 80°C

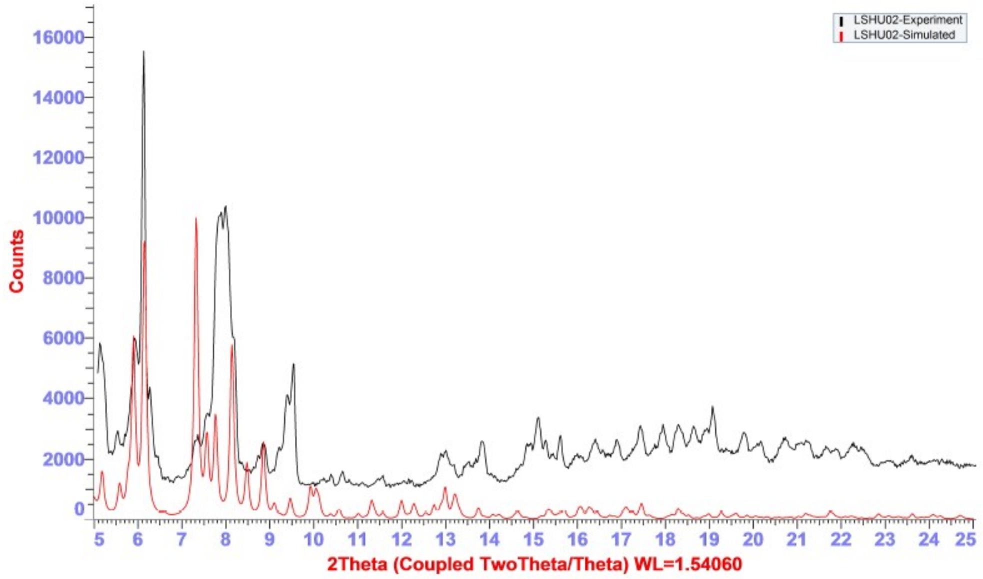

Fig. S10 Powder XRD patterns of **LSHU02** shows that the crystals can maintain crystallinity at ambient. The dramatically difference diffraction properties of **LSHU02** compared to **LSHU01** may attributed to different solvent molecule species and quantity as they were obtained in different v/v $CHCl_3$-CH_3OH mixtures and this is also accordance with single crystals X-ray diffraction experiments .

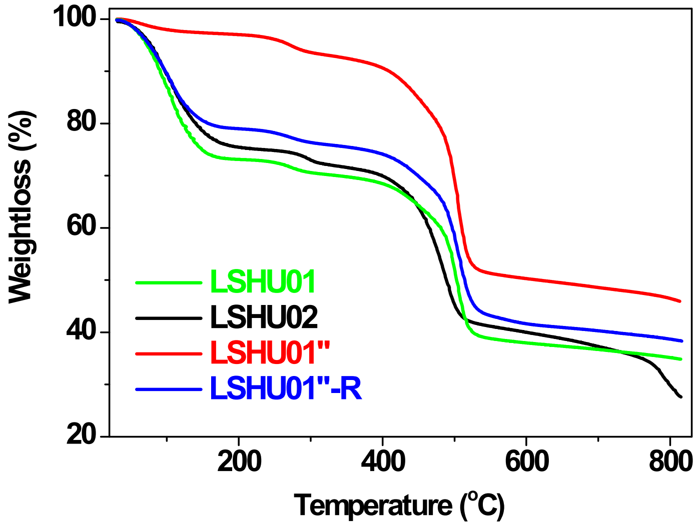

Fig. S11 TGA (heating rate 15 °C/min in N_2) profiles of **LSHU01** (as synthesized), **LSHU01"** (activated at 150°C), **LSHU01"-R** (resolvated), and **LSHU02** (as synthesized), respectively. For **LSHU01, LSHU02,** and **LSHU01"-R**, TGA analysis indicates that the onset of the solvent loss is at the very beginning of recording and the weight decreases sharply corresponding to the release of the solvent $CHCl_3$ and/or CH_3OH molecules. Further weight loss takes place at ca. 200°C which can be assigned to lose coordinated water molecules and Cl atoms (see TPD experiment above for details). And then the framework of the compounds began to decompose gradually without a stable weight below 800 °C. TGA measurements revealed that Co crystals can be evacuated (at 150 °C for three hours) and resolvated (v : v= 1:1, CH_3OH and $CHCl_3$ mixture for three days). For the activated samples of **LSHU01"**, the weight loss (< 3%) from room temperature to 150 °C may reasoned to the absorption of substances in air and the observation of weight loss signals at ca. 200 °C further confirmed the stability of the overall cage structure.

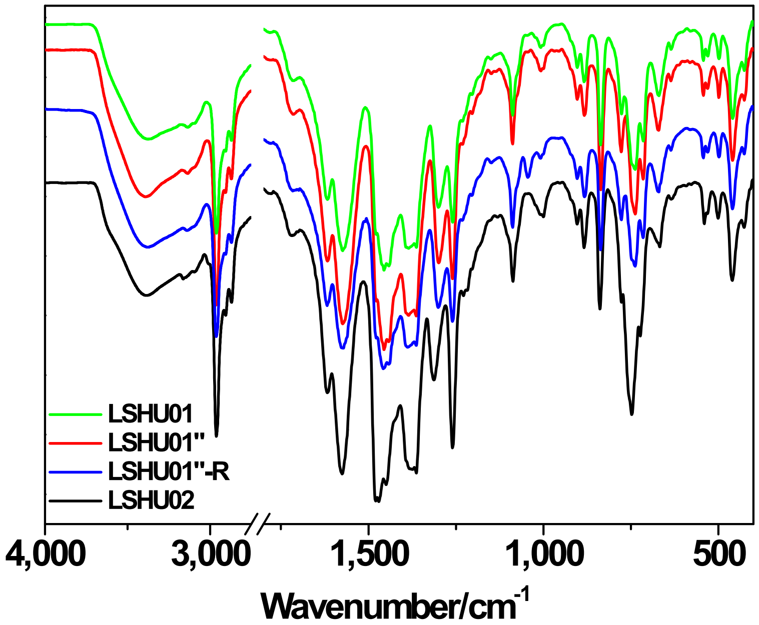

Fig. S12 FT-IR spectra of **LSHU01** (as synthesized), **LSHU01''** (activated), **LSHU01''-R** (Resolvated), and **LSHU02** (as synthesized) showing the similar structures and stability of Co_{48} cages.

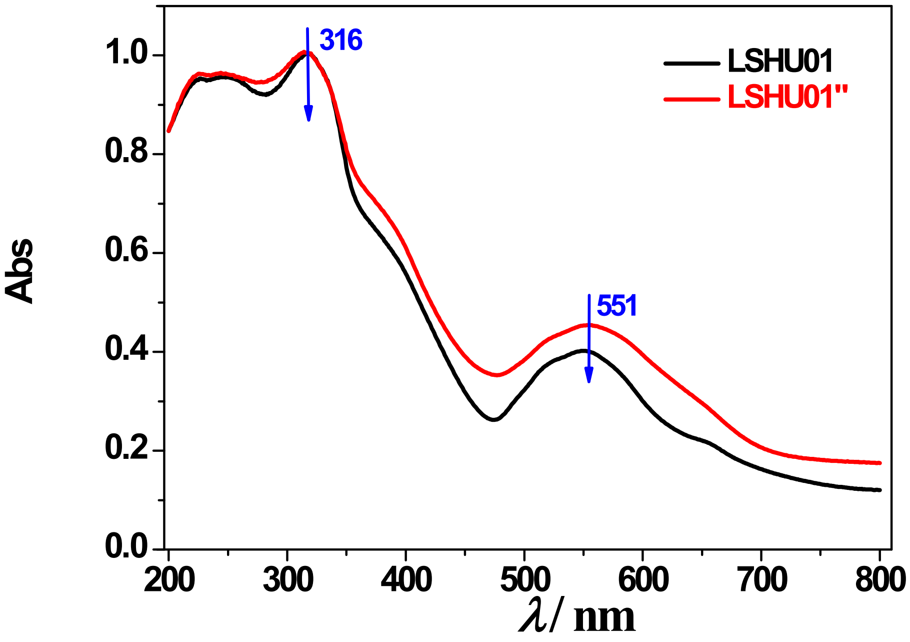

Fig. S13 UV-Vis spectra of **LSHU01** (as synthesized) and **LSHU01''** (activated) showing stability of Co_{48} cages.

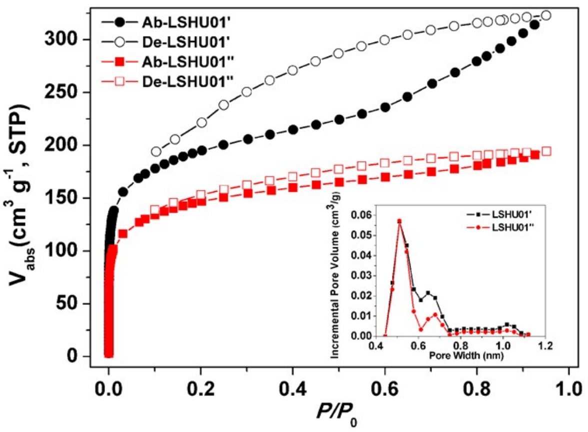

Fig. S14 Argon sorption isotherms (87 K) on LSHU01 evacuated at 353K and 423K; Inset: The pore size distribution calculated by the DFT method.

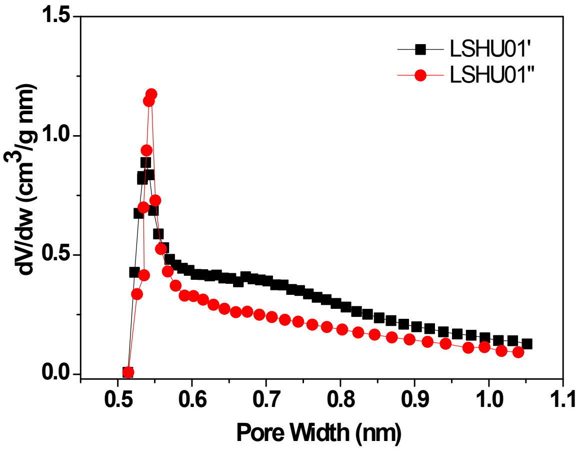

Fig. S15 The pore size distribution calculated by the HK methods.

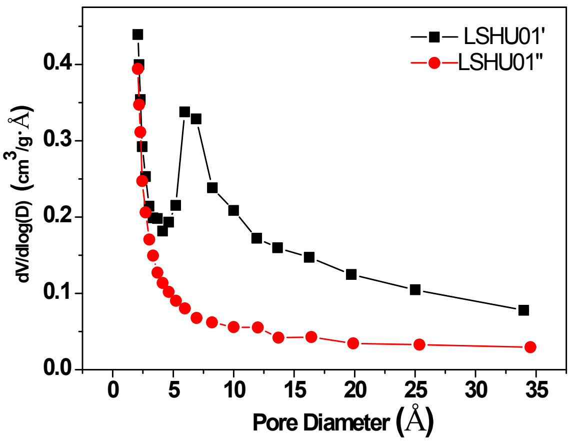

Fig. S16 The pore size distribution calculated by the BJH methods.

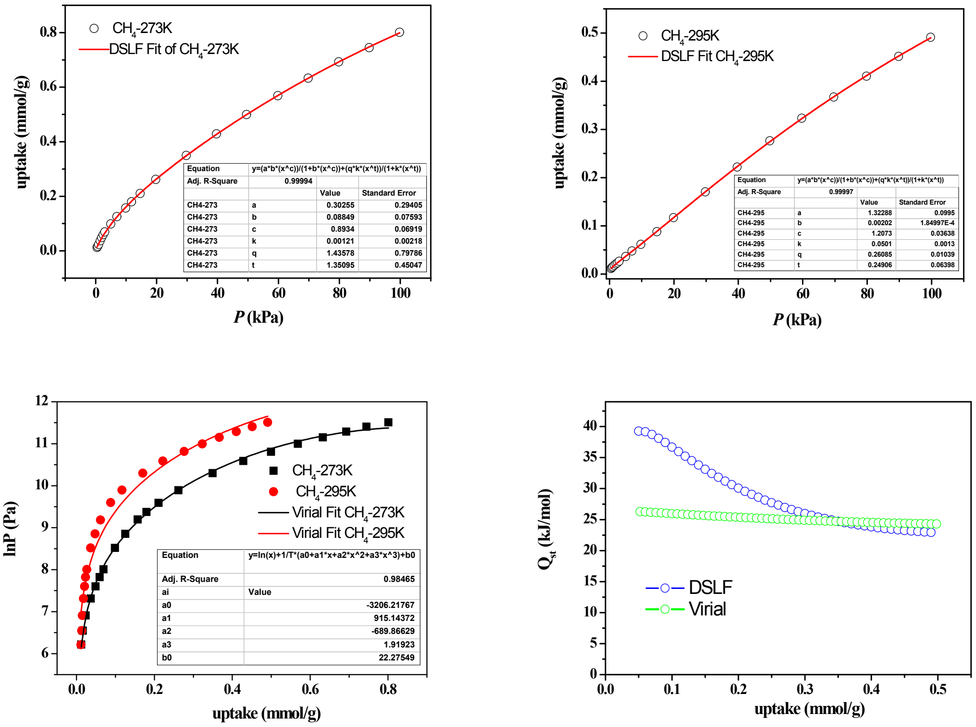

Fig. S17 DSLF (up-left 273K, up-right-295K) and Virial (bottom-left) fit of CH_4 adsorption isotherms of **LSHU01'** and the Q_{st} of CH_4 obtained by DSLF and Virial fitting equations (bottom-right).

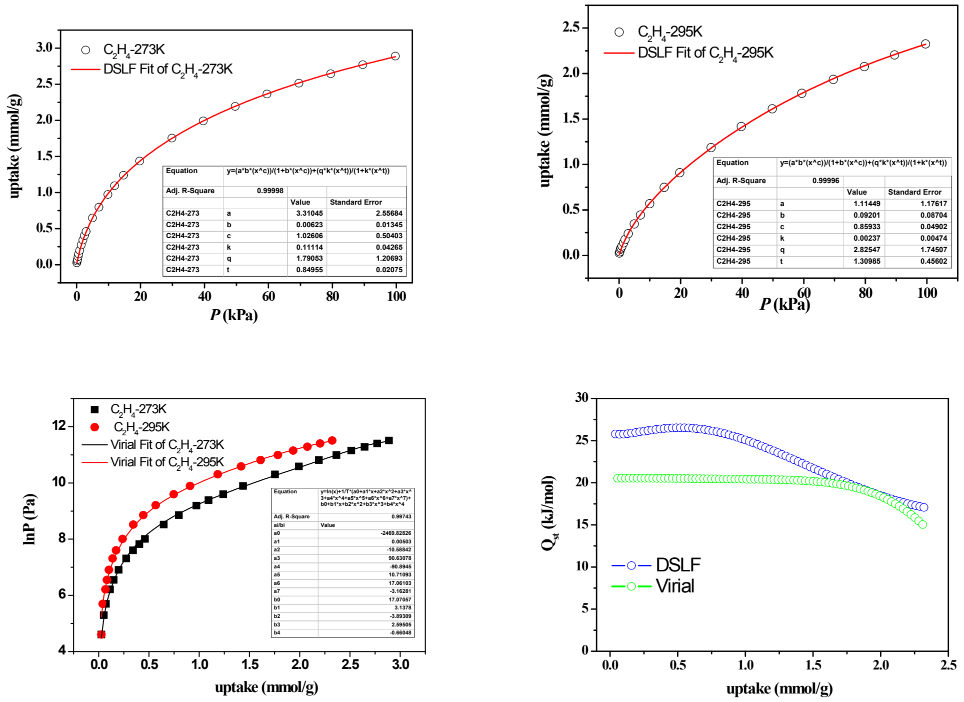

Fig. S18 DSLF (up-left 273K, up-right-295K) and Virial (bottom-left) fit of C_2H_4 adsorption isotherms of **LSHU01'** and the Q_{st} of C_2H_4 obtained by DSLF and Virial fitting equations (bottom-right).

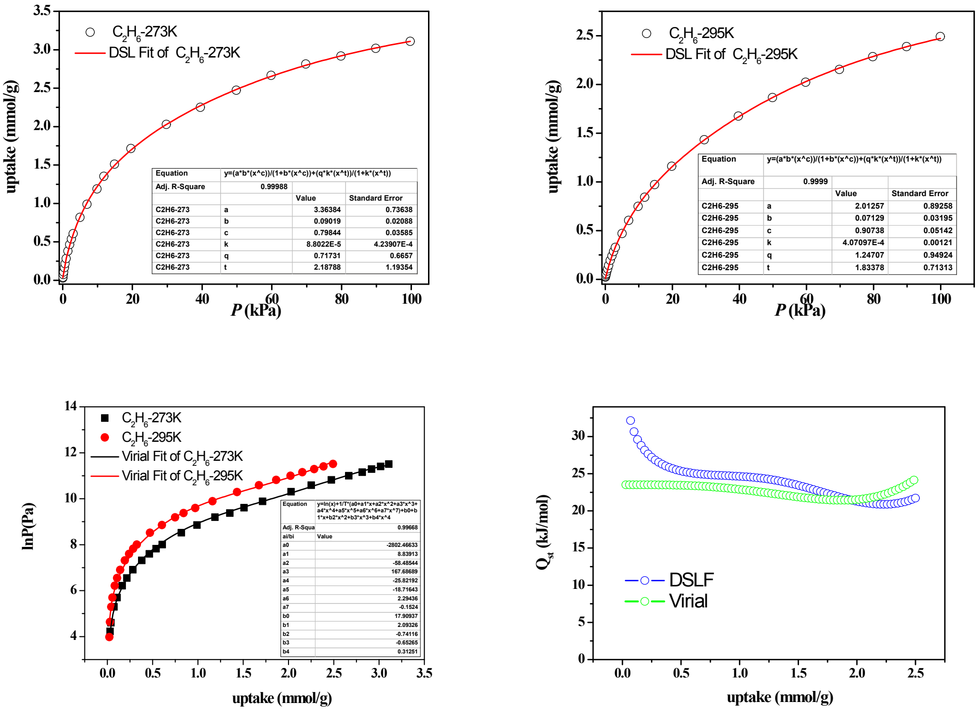

Fig. S19 DSLF (up-left 273K, up-right-295K) and Virial (bottom-left) fit of C_2H_6 adsorption isotherms of **LSHU01'** and the Q_{st} of C_2H_6 obtained by DSLF and Virial fitting equations (bottom-right).

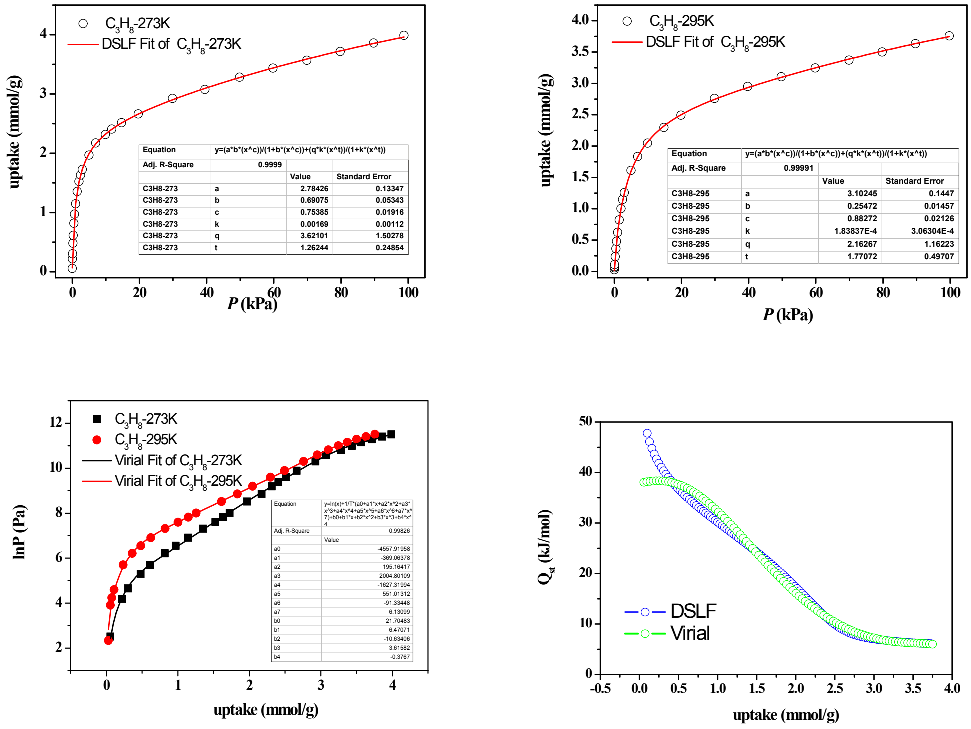

Fig. S20 DSLF (up-left 273K, up-right-295K) and Virial (bottom-left) fit of C_3H_8 adsorption isotherms of **LSHU01'** and the Q_{st} of C_3H_8 obtained by DSLF and Virial fitting equations (bottom-right).

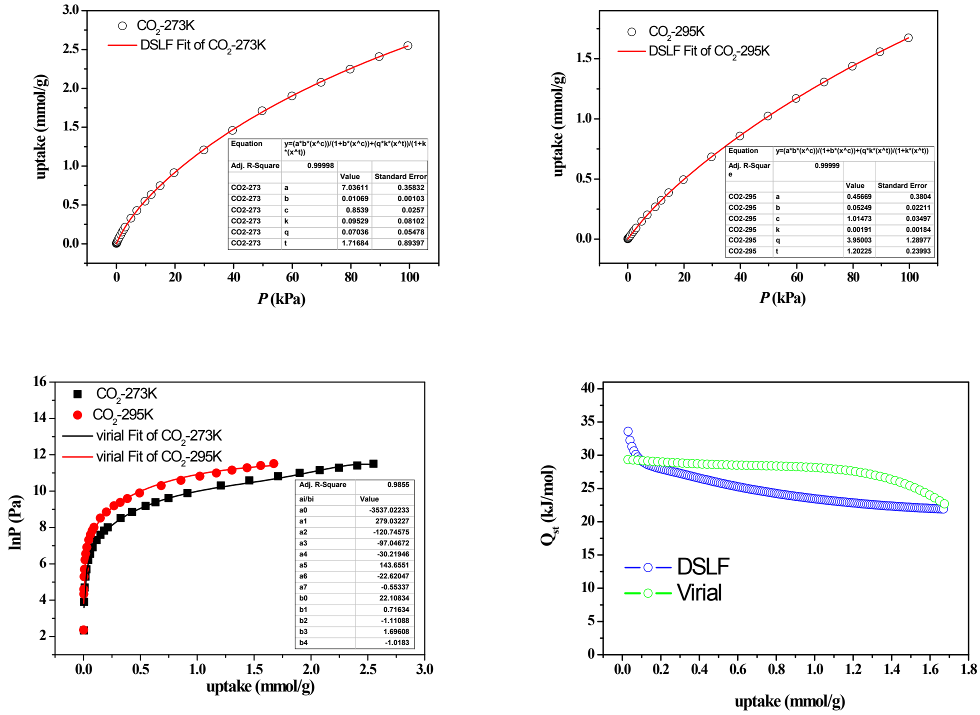

Fig. S21 DSLF (up-left 273K, up-right-295K) and Virial (bottom-left) fit of CO_2 adsorption isotherms of **LSHU01'** and the Q_{st} of CO_2 obtained by DSLF and Virial fitting equations (bottom-right).

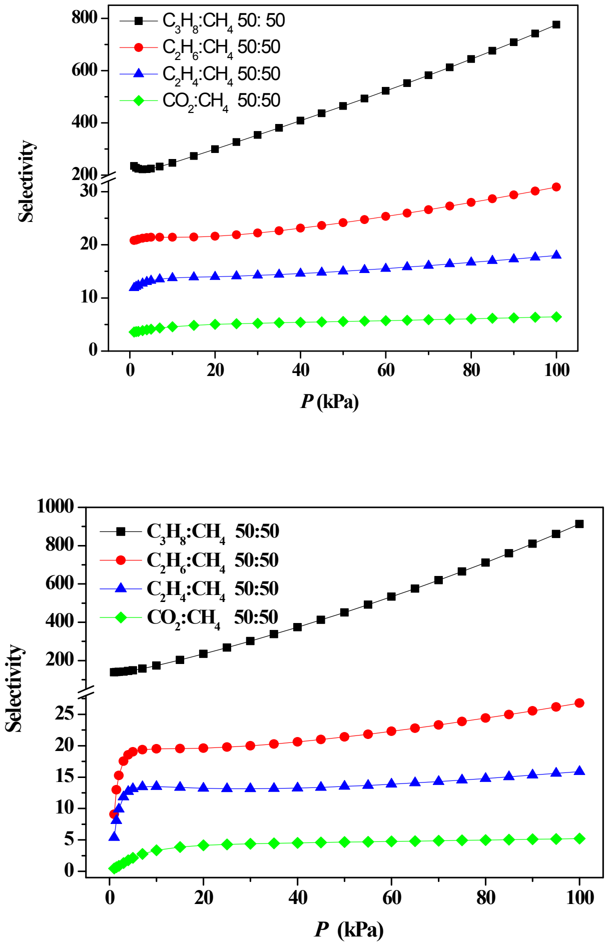

Fig. S22 Gas mixture adsorption selectivity predicted by IAST for **LSHU01'** at 273K (up) and 295K (bottom).

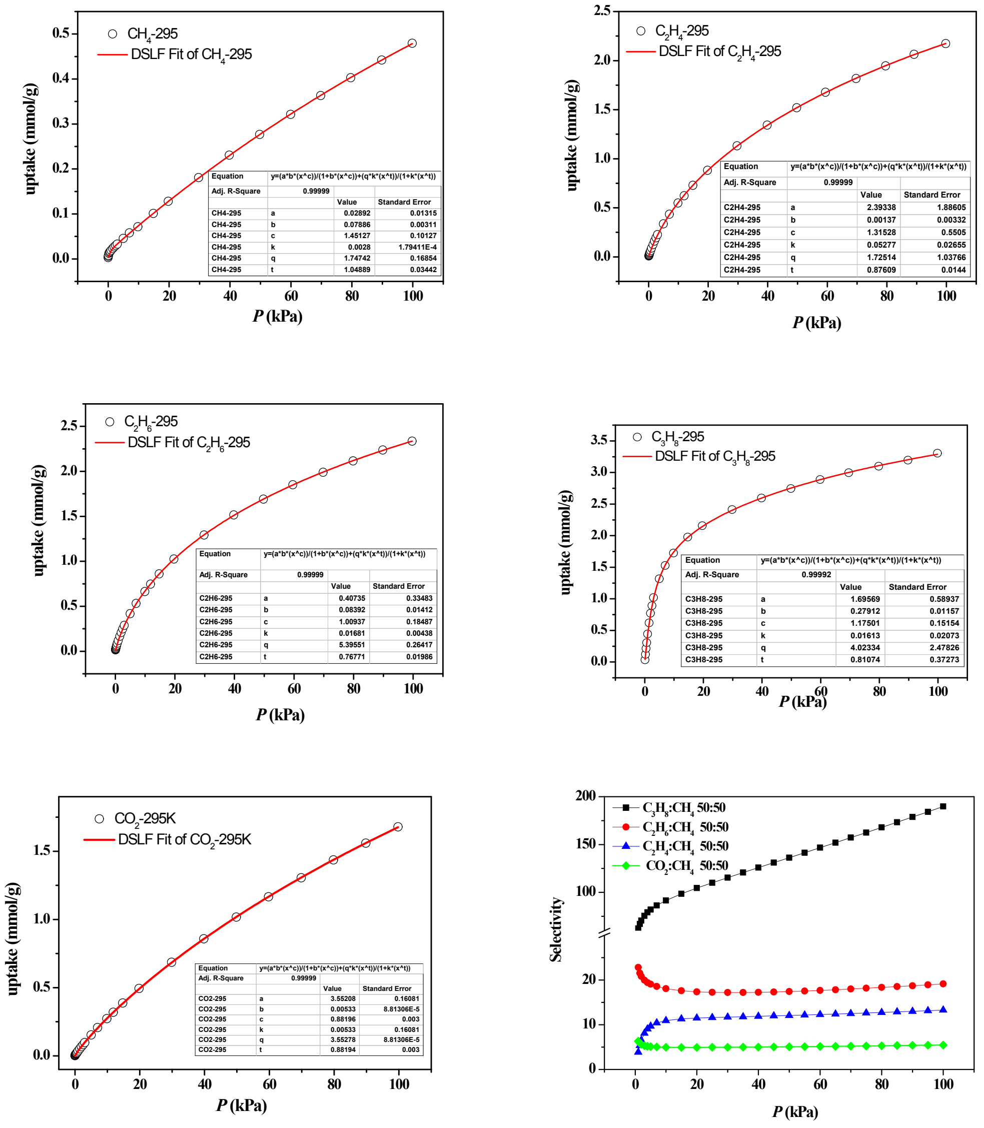

Fig. S23 Gas adsorption isotherms, DSLF fit of **LSHU01''** at 295K and gas mixture adsorption selectivity predicted by IAST for **LSHU01''** at 295K.

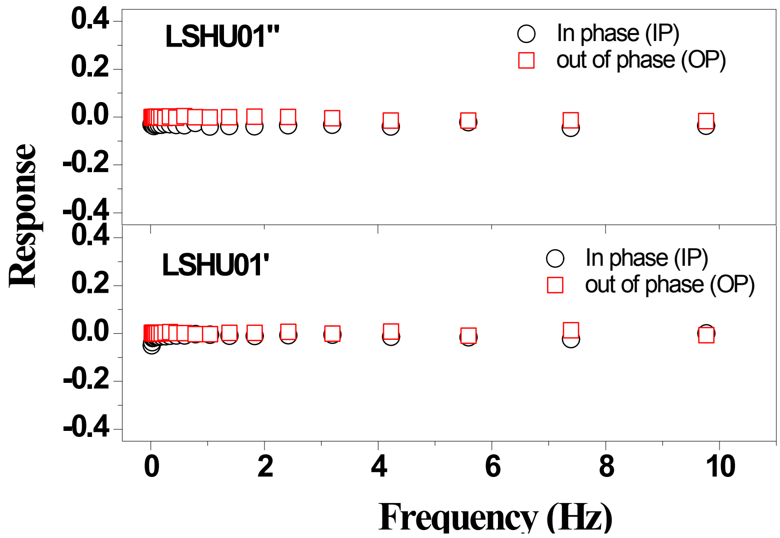

Fig. S24 Frequency response (FR) spectra of CH_4 for **LSHU01'** (bottom) and **LSHU01"** (up)

Estimation of the isosteric heats of gas adsorption

Isosteric heats are usually calculated by IAST method by fitting adsorption isotherms with virial-type expression at two temperatures [S2,S3].

$$lnP = LnN + \frac{1}{T}\sum_{i=0}^{m} a_i N^i + \sum_{i=0}^{n} b_i N^i \qquad \text{(Virial)}$$

Here, P is the pressure expressed in Pa, N is the amount adsorbed in mmol/g, T is the temperature in K, a_i and b_i are virial coefficients, and m, n represent the number of coefficients required to adequately describe the isotherms (m and n were gradually increased until the contribution of extra added a and b coefficients was deemed to be statistically insignificant towards the overall fit, and the average value of the squared deviations from the experimental values was minimized). The values of the virial coefficients a_0 through a_m were then used to calculate the isosteric heat of adsorption using the following expression:

$$Q_{st} = -R\sum_{i=0}^{m} a_i N^i$$

Considering the complicated multi-microporous structures and the evacuation temperature-dependent Ar absorption properties of **LSHU01'** and **LSHU01"**, and also due to the fact that relative lower R^2 (98.5%-99.8%) were obtained by fitting the sorption data with virial-equation, isosteric heats (Q_{st}) for CO_2, CH_4, C_2H_4, C_2H_6 and C_3H_8 were also calculated by fitting absorption isotherms with Dual-site Langmuir Freundlich (DSLF) equation for comparison, which resulted good R^2(>0.9999, see Figures below).

$$q = \frac{a * b * P^c}{1 + b * P^c} + \frac{q * k * P^t}{1 + k * P^t} \qquad \text{(DSLF)}$$

P is the pressure of the bulk gas at equilibrium with the adsorbed phase (kPa), q is the adsorbed amount per mass of adsorbent (mmol/g), a and q are the saturation capacities of sites 1 and 2 (mmol/g), b and k are the affinity coefficients of sites 1 and 2 (1/kPa), and c and t represent the deviations from an ideal homogeneous surface. R is the universal gas constant. The DSLF fitted parameters can be used calculated isosteric heat of adsorption using the Clausius–Clapeyron relation and also were used to predict multi-component adsorption with IAST.[S4]

$$Q_{st} = R\frac{T_1T_2}{T_2 - T_1}(lnP_2 - lnP_1)_q$$

PLATON SQUEEZE Result

The *PLATON* SQUEEZE results as follows:

For **LSHU01:**

```
# Note: Data are Listed for all Voids in the P1 Unit Cell
# i.e. Centre of Gravity, Solvent Accessible Volume,
# Recovered number of Electrons in the Void and
# Details about the Squeezed Material
loop_
  _platon_squeeze_void_nr
  _platon_squeeze_void_average_x
  _platon_squeeze_void_average_y
  _platon_squeeze_void_average_z
  _platon_squeeze_void_volume
  _platon_squeeze_void_count_electrons
  _platon_squeeze_void_content
   1 -0.022 -0.021 -0.001     54243      14993 ' '
   2  0.591  0.013  0.168        10          2 ' '
   3  0.755  0.075  0.835        12          2 ' '
   4  0.483  0.066  0.112         6          1 ' '
   5  0.346  0.088  0.498        11          1 ' '
   6  0.267  0.084  0.778         7          2 ' '
   7  0.750  0.150  0.555         6          2 ' '
   8  0.916  0.184  0.778         7          1 ' '
   9  0.321  0.245  0.835        12          3 ' '
  10  0.912  0.258  0.498        11          1 ' '
  11  0.399  0.250  0.555         6          2 ' '
  12  0.184  0.267  0.222         7          2 ' '
  13  0.075  0.321  0.165        12          1 ' '
  14  0.258  0.346  0.502        11          3 ' '
  15  0.421  0.409  0.168        11          2 ' '
  16  0.150  0.399  0.445         6          1 ' '
  17  0.934  0.417  0.112         7          2 ' '
  18  0.013  0.421  0.832        11          1 ' '
  19  0.417  0.483  0.888         6          2 ' '
  20  0.583  0.517  0.112         7          1 ' '
  21  0.987  0.579  0.168        11          3 ' '
  22  0.579  0.591  0.832        11          1 ' '
  23  0.066  0.583  0.888         6          2 ' '
```

24	0.850	0.601	0.555	7	2 ' '
25	0.742	0.654	0.498	12	1 ' '
26	0.925	0.679	0.835	11	3 ' '
27	0.088	0.742	0.502	11	2 ' '
28	0.816	0.733	0.778	6	1 ' '
29	0.679	0.755	0.165	11	1 ' '
30	0.601	0.750	0.445	7	2 ' '
31	0.084	0.816	0.222	6	2 ' '
32	0.250	0.850	0.445	7	1 ' '
33	0.654	0.912	0.502	11	3 ' '
34	0.245	0.925	0.165	11	1 ' '
35	0.733	0.916	0.222	6	2 ' '
36	0.517	0.934	0.888	6	2 ' '
37	0.409	0.987	0.832	11	1 ' '

For **LSHU02:**

Note: Data are Listed for all Voids in the P1 Unit Cell

i.e. Centre of Gravity, Solvent Accessible Volume,

Recovered number of Electrons in the Void and

Details about the Squeezed Material

loop_

_platon_squeeze_void_nr

_platon_squeeze_void_average_x

_platon_squeeze_void_average_y

_platon_squeeze_void_average_z

_platon_squeeze_void_volume

_platon_squeeze_void_count_electrons

_platon_squeeze_void_content

1 -0.008 -0.011 -0.001 71372 14690 '

According to the SQUEEZE analysis and TGA experiments, the TENTATIVE formula for the compounds with the solvents might be:

$[M_{48}(TC4A)_{12}(L)_{18}Cl_{12}(H_2O)_6]\ (CH_3OH)_x(CHCl_3)_y$

Based on molecular electrons: x= 288, y=0 or x=0, y= 86 for **LSHU01** (**1**)

x= 272, y=0 or x=0, y= 84 for **LSHU02** (**2**)

(per CH_3OH: 18e^-; per $CHCl_3$: 58e^-)[S5]

Based on molecular volume: x= 153, y=0 or x=0, y= 75 for **LSHU01** (**3**)

x= 202, y=0 or x=0, y= 99 for **LSHU02** (**4**)

(per CH_3OH: 108 Å^3; $CHCl_3$: 240 Å^3) [S6]

Based on TGA: x= 198, y=0 or x=0, y= 56 for **LSHU01** (**5**)

x= 179, y=0 or x=0, y= 50 for **LSHU02** (**6**)

(27% weight loss for **LSHU01** and 24.5% for **LSHU02** asigned to solvents)

Combination of SQUEEZE analysis and TGA experiments, it is more reasonable to give possible formula as that in (**5**) and (**6**) for **LSHU01** and **LSHU02,** respectively.

Table S1 Crystal data and structure refinement for **LSHU01** and **LSHU02**

	**LSHU01**	**LSHU02**
formula*	$C_{642}H_{612}Cl_{12}Co_{48}N_{72}O_{126}S_{48}$	$C_{642}H_{612}Cl_{12}Ni_{48}N_{72}O_{126}S_{48}$
formula wt.	16144.92	16134.36
Cryst. syst	*Trigonal*	*Trigonal*
space group	*R-3*	*R-3*
a (Å)	37.232(8)	37.799(6)
b (Å)	37.232(8)	37.799(6)
c (Å)	80.093(18)	86.192(13)
α (°)	90	90
β (°)	90	90
γ (°)	120	120
V (Å^3)	96150(46)	106652(36)
Z	3	3
D_c/g cm^{-3}*	0.836	0.754
μ/mm^{-1}	0.747	0.749
F(000)	24732	24876
Tot. Data	78546	65176
Uniq. Data	25993	22657
R_{int}	0.040	0.101
GOF	0.98	1.03
$R_1{}^a$ [I>2σ(I)]	0.0735	0.0617
$wR_2{}^b$(all data)	0.2227	0.1692

$^aR_1 = \Sigma||F_0|-|F_c||/\Sigma|F_0|$; $^bwR_2 = \{\Sigma[w(F_0{}^2-F_c{}^2)^2]/\Sigma[w(F_0{}^2)^2]\}^{1/2}$

* The formula and D_c is not included the unidentified disordered solvents

Table S2. Selected bond distances (Å) and BVS calculations for **LSHU01** and **LSHU02**

**LSHU01**				**LSHU02**			
Band	Distance	r	Value	Band	Distance	r	Value
Co(1)–S(1)	2.483(3)	2.06	0.162	Ni(1)–S(1)	2.464(3)	2.04	0.318
Co (1)–O(1)	1.980(5)	1.692	0.459	Ni (1)–O(1)	1.931(5)	1.654	0.474
Co (1)–O(4)	2.001(5)	1.692	0.435	Ni (1)–O(4)	1.971(6)	1.654	0.425
Co (1)–O(9)	2.085(5)	1.692	0.346	Ni (1)–O(9)	2.012(6)	1.654	0.380
Co (1)–N(12)	2.112(7)	1.84	0.479	Ni (1)–O(17)	1.949(6)	1.654	0.451
Co (1)–Cl(2)	2.6811(4)	2.01	0.162	Ni (1)–Cl(2)	2.731(4)	2.02	0.146
valence			2.200	valence			2.194
Co (2)–S(2)	2.474(2)	2.06	0.327	Ni (2)–S(2)	2.469(3)	2.04	0.314
Co (2)–O(1)	2.011(5)	1.692	0.422	Ni (2)–O(1)	1.959(6)	1.654	0.439
Co (2)–O(2)	1.954(5)	1.692	0.493	Ni (2)–O(2)	1.985(6)	1.654	0.409
Co (2)–O(10)	1.946(6)	1.692	0.503	Ni (2)–O(10)	2.000(6)	1.654	0.394
Co (2)–O(13)	2.028(4)	1.692	0.403	Ni (2)–O(13)	1.976(6)	1.654	0.420
Co (2)–Cl(2)	2.6703(6)	2.01	0.168	Ni (2)–Cl(2)	2.583(3)	2.02	0.218
valence			2.316	valence			2.193
Co (3)–S(3)	2.541(3)	2.06	0.273	Ni (3)–S(3)	2.440(3)	2.04	0.339
Co (3)–O(2)	1.980(5)	1.692	0.459	Ni (3)–O(2)	1.985(6)	1.654	0.410
Co (3)–O(3)	1.965(4)	1.692	0.478	Ni (3)–O(3)	2.018(5)	1.654	0.374
Co (3)–O(14)	1.998(4)	1.692	0.437	Ni (3)–O(14)	2.006(6)	1.654	0.386
Co (3)–O(20)[a]	2.014(5)	1.692	0.419	Ni (3)–O(21)	2.089(8)	1.654	0.309
Co (3)–Cl(2)	2.8134(6)	2.01	0.134	Ni (3)–Cl(2)	2.583(3)	2.02	0.218
valence			2.200	valence			2.036
Co (4)–S(4)	2.523(2)	2.06	0.286	Ni (4)–S(4)	2.427(3)	2.04	0.351
Co (4)–O(3)	1.992(4)	1.692	0.446	Ni (4)–O(3)	2.021(5)	1.654	0.370
Co (4)–O(4)	2.029(5)	1.692	0.403	Ni (4)–O(4)	2.006(7)	1.654	0.386
Co (4)–O(21)	2.084(6)	1.692	0.347	Ni (4)–O(18)	2.045(5)	1.654	0.349
Co (4)–O(19)[b]	2.044(7)	1.692	0.386	Ni (4)–N(8)	2.088(14)	1.75	0.400
Co (4)–Cl(2)	2.6088(5)	2.01	0.198	Ni (4)–Cl(2)	2.665(3)	2.02	0.175
valence			2.066	valence			2.031
Co (5)–S(5)	2.483(2)	2.06	0.319	Ni (5)–S(5)	2.431(3)	2.04	0.348
Co (5)–O(5)	1.984(4)	1.692	0.454	Ni (5)–O(5)	2.001(5)	1.654	0.391
Co (5)–O(8)	1.982(4)	1.692	0.457	Ni (5)–O(8)	1.992(5)	1.654	0.401
Co (5)–O(18)[b]	2.073(5)	1.692	0.356	Ni (5)–O(11)[d]	2.054(7)	1.654	0.339

Co (5)–N(8)[c]	2.105(10)	1.84	0.491	Ni (5)–N(12)[e]	2.035(12)	1.75	0.460
Co (5)–Cl(1)	2.6071(5)	2.01	0.199	Ni (5)–Cl(1)	2.713(3)	2.02	0.154
valence			2.276	valence			2.093
Co (6)–S(6)	2.4963(19)	2.06	0.307	Ni (6)–S(6)	2.448(3)	2.04	0.332
Co (6)–O(5)	2.020(4)	1.692	0.412	Ni (6)–O(5)	2.013(5)	1.654	0.379
Co (6)–O(6)	1.978(5)	1.692	0.462	Ni (6)–O(6)	1.990(5)	1.654	0.403
Co (6)–O(15)	2.095(5)	1.692	0.337	Ni (6)–O(15)	2.081(7)	1.654	0.315
Co (6)–N(4)	2.066(6)	1.84	0.543	Ni (6)–N(4)	2.076(7)	1.75	0.414
Co (6)–Cl(1)	2.6943(4)	2.01	0.157	Ni (6)–Cl(1)	2.592(3)	2.02	0.214
valence			2.219	valence			2.058
Co (7)–S(7)	2.510(2)	2.06	0.297	Ni (7)–S(7)	2.483(3)	2.04	0.302
Co (7)–O(6)	1.981(4)	1.692	0.458	Ni (7)–O(6)	1.958(6)	1.654	0.439
Co (7)–O(7)	1.980(4)	1.692	0.459	Ni (7)–O(7)	1.952(5)	1.654	0.447
Co (7)–O(16)	1.988(4)	1.692	0.449	Ni (7)–O(16)	1.972(6)	1.654	0.423
Co (7)–O(12)	2.014(6)	1.692	0.418	Ni (7)–O(19)[d]	2.042(8)	1.654	0.352
Co (7)–Cl(1)	2.7176(5)	2.01	0.147	Ni (7)–Cl(1)	2.716(3)	2.02	0.152
valence			2.228	valence			2.116
Co (8)–S(7)	2.532(2)	2.06	0.279	Ni (8)–S(8)	2.487(3)	2.04	0.299
Co (8)–O(7)	1.999(4)	1.692	0.437	Ni (8)–O(7)	1.952(5)	1.654	0.447
Co (8)–O(8)	1.996(4)	1.692	0.440	Ni (8)–O(8)	1.985(6)	1.654	0.409
Co (8)–O(11)[b]	2.017(6)	1.692	0.415	Ni (8)–O(20)[d]	1.998(8)	1.654	0.395
Co (8)–O(17)[b]	2.010(6)	1.692	0.423	Ni (8)–O(12)[e]	1.973(6)	1.654	0.422
Co (8)–Cl(1)	2.7171(5)	2.01	0.148	Ni (8)–Cl(1)	2.728(3)	2.02	0.147
valence			2.143	valence			2.119

a: 1-y,1+x-y,z; b: -x+y,1-x,z; c: 2/3+x-y,1/3+x,4/3-z; d: -x+y,1-x,z; e: -1/3+y,1/3-x+y,4/3-z;

Table S3. Comparison on gas separation performances of some coordination compounds.

Compounds	Selectivity(at ambient)				Refs.
	C_3H_8/CH_4	C_2H_6/CH_4	C_2H_4/CH_4	CO_2/CH_4	
Co_{48} cage (LSHU01')	**912.6**	**26.8**	**15.9**	**5.2**	This work
CIAC-124 (nanotube)	~25	10	-	3.6	*Dalton Trans.,* 2018, **47**, 1782–1785
CIAC-115 (Ni_{40} cage)	~30	8.3	-	3.0	*J. Am. Chem. Soc*., 2016, **138**, 2969-2972
FIR-7a-ht (Zn-MOF)	78.8	14.6	8.6	-	*Chem. Commun.,* 2013, **49**, 11323-11325
UTSA-35a (Cd-MOF)	~ 80	<15	8	-	*Chem. Commun.,* 2012, **48**, 6493–6495
JLU-Liu5 (In-MOF)	107.8	17.6	-	-	*Chem. Commun.,* 2014, **50**, 8648–8650
JLU-Liu6(Zn-MOF)	274.6	20.4	-	-	
JLU-Liu18(In-MOF)	108.2	13.1	-	5.4	*J. Mater. Chem. A,* 2015, **3**, 16627–16632
JLU-Liu7(In-TCPP-MOF)	128.5	50.4	23.3	6.9	*Inorg. Chem. Front.,* 2017, **4,** 139–143
JLU-Liu22(Cu-MOF)	271.5	14.4	-	9.4	*Chem. Commun.,* 2015, **51**, 15287–15289
JLU-Liu15(Cu-MOF)	461.5	27.8	-	3.05	*Cryst. Growth Des.,* 2015, **15**, 4901–4907

References

S1. Y. F. Bi, X. T. Wang, W. P. Liao, X. F. Wang, X. W. Wang, H. J. Zhang and S. Gao, *J. Am. Chem. Soc.*, 2009, **131**, 11650-11651.

S2. J. L. C. Rowsell and O. M. Yaghi, *J. Am. Chem. Soc.*, 2006, **128**, 1304-1315.

S3. L. T. Du, Z. Y. Lu, K. Y. Zheng, J. Y. Wang, X. Zheng, Y. Pan, X. Z. You and J. F. Bai, *J. Am. Chem. Soc.*, 2013, **135**, 562-565.

S4. T. Runčevski, M. T. Kapelewski, R. M. Torres-Gavosto, J. D. Tarver, C. M. Brown and J. R. Long, *Chem. Commun.*, 2016, **52**, 8251-8254.

S5. B. E. Poling, J. M. Prausnitz and J. P. ÓConnell, *The Properties of Gases and Liquids*, McGraw-Hill, New York, 5th edn, 2001.

S6. D. R. Lide, *CRC Handbook of Chemistry and Physics*, CRC Press, 88th edn, 2007.
